# The Interplay Between Therapeutic Self-Amplifying RNA and the Innate Immune System: Balancing Efficiency and Reactogenicity

**DOI:** 10.3390/ijms26188986

**Published:** 2025-09-15

**Authors:** Dmitry Kunyk, Marina Plotnikova, Mikhail Bespalov, Daniil Shevyrev, Sergey Klotchenko, Roman Ivanov, Vasiliy Reshetnikov

**Affiliations:** 1 Translational Medicine Research Center, Sirius University of Science and Technology, 354340 Sochi, Russia; 2 Smorodintsev Research Institute of Influenza, The Ministry of Health of the Russian Federation, 197022 Saint-Petersburg, Russiafosfatik@mail.ru (S.K.)

**Keywords:** saRNA, self-amplifying RNA, therapeutics, vaccine, reactogenicity, optimization, immunity, RLR, replicon, VEEV, clinical development

## Abstract

Self-amplifying RNA (saRNA) is a promising platform for the production of vaccines, anti-tumor therapeutics, and gene therapy solutions. One of the advantages of the saRNA platform is the ability to use small doses of the therapeutic while maintaining prolonged expression of the target protein. However, the presence of auxiliary sequences encoding non-structural alphavirus proteins, which facilitate the replication of saRNA in cells, necessitates a thorough assessment of the biosafety of this platform. In our review, we focus on saRNA functions in the context of its interaction with the innate immune system. Firstly, an analysis is conducted of the side effects of candidate saRNA therapeutics, as observed in preclinical and clinical trials. Then, the mechanisms underlying the function of saRNA products derived from various alphavirus genomes in cell systems are discussed, as well as the reasons for their reactogenicity. The key approaches to optimizing the saRNA platform, which are aimed at reducing the activation of the innate immune response and cytopathic effects, are described. To summarize, this review enables us to systematize our knowledge on the advantages and disadvantages of saRNA, as well as potential approaches to improving this platform in order to develop more effective and safer therapeutics.

## 1. Introduction

RNA-based therapeutics have great potential as both therapeutic and preventive solutions. Currently, an increasing number of studies are focused on the development of RNA vaccines, anti-tumor therapeutics, and RNA-based gene therapy products. Key advantages of RNA platforms include flexible manufacturing technologies, rapid development, and relatively low cost. The role of messenger RNA (mRNA) in protein biosynthesis was discovered in 1961 [1], and research since then has been focused on studying the structural and functional properties of this molecule. In 1990, Wolff et al. demonstrated that the direct administration of mRNA encoding chloramphenicol acetyltransferase into mouse muscle resulted in the expression of this enzyme [2]. This discovery served as a catalyst for the development of mRNA-based gene therapy and vaccination technologies.

However, a significant breakthrough in mRNA therapeutic technology was achieved by Katalin Karikó, who, in 2008, demonstrated that introducing a modified pseudouridine nucleoside into mRNA reduced innate immune response activation by decreasing interactions with the pattern recognition receptors (PRRs) [3]. The second key milestone for RNA-based therapy was the discovery of an effective delivery system based on lipid nanoparticles and its approval for therapeutic use by the FDA in 2012 [4]. These discoveries served as a starting point for the development of the first mRNA vaccines, RNA-1273 (Moderna, Cambridge, MA, USA) and BNT162b2 (Pfizer (New York, NY, USA)/BioNTech (Mainz, Germany)), which successfully passed clinical trials during the COVID-19 pandemic and were approved in 2020 and 2021 [5,6]. The success of these vaccines motivated researchers to develop new mRNA-based therapeutics and improve delivery systems for targeting mRNA therapeutics to specific organs [7]. Currently, numerous preclinical studies have been conducted using in vitro transcribed RNA (IVT RNA) for anti-tumor therapy, vaccines against infectious diseases, and gene therapy [8,9].

However, despite the proven effectiveness of mRNA therapeutics, there are several issues that limit their application. mRNA molecules are short-lived and are susceptible to degradation by cellular exonucleases. The target effect requires relatively high doses of the therapeutic, which can lead to undesirable events due to the potential reactogenicity of the mRNA itself and the lipids used in nanoparticles [10,11,12]. Moreover, several studies have demonstrated a rapid decline in virus-neutralizing antibody titers following immunization with mRNA vaccines [13]. Thus, further optimization of the mRNA platform is needed to enhance the efficacy and safety of RNA therapeutics. One of the most promising approaches is the development of therapeutics based on self-amplifying RNA (saRNA) [14].

Self-amplifying RNA (saRNA) is a unique class of RNA molecule that exhibits the capacity to increase its copy number within the cell, a property that distinguishes it from conventional mRNA molecules. The amplification process of saRNA results in the formation of antisense RNA complementary to sense RNA, which forms a duplex. saRNA contains sequences from the replication machinery of alphaviruses that encode non-structural proteins in addition to the gene of interest (transgene). The initially translated RNA replicon of alphaviruses facilitates subsequent transcription, replication, and high expression of the target protein. Recent studies have shown that saRNA and the encoded protein can be detected at the injection site (muscle) and in the lymph nodes and spleen for up to 60 days post-administration [15]. Components of the therapeutics based on classical linear mRNA have a shorter elimination half-life [16]. The amplification capability of saRNA potentially allows for reducing the dose of the administered therapeutic sufficient to elicit adaptive and protective immunity, thereby decreasing the possible reactogenicity of the mRNA therapeutic. Moreover, reducing the dose of the administered therapeutic will help to lower production costs.

However, the use of saRNA-based therapeutics remains limited due to the potential side effects associated with the increased activation of the innate immune response [17]. Currently, there is only one approved saRNA-based therapeutic: ARCT-154 against SARS-CoV-2 from Arcturus Therapeutics [18]. Transgene replication is accompanied by the formation of an intermediate replication product, double-stranded RNA (dsRNA). dsRNA and other replication products are classified as pathogen-associated molecular patterns (PAMPs) that can activate the innate immune receptors and trigger a robust immune response. Furthermore, the non-structural proteins of alphaviruses can directly affect cell viability. Vanluchene et al. [19] showed that increasing the dose of saRNA encoding GFP to 0.1 µg promotes the cytopathic effect on retinal pigment epithelial cells ARPE-19 and the human Müller cell line.

Despite the growing number of studies on saRNA applications, its function and interaction with the immune system [20,21], the development of a safe therapeutic saRNA platform requires new strategies to optimize the viral replication machinery exploited by saRNA.

In our review, we focused on the fundamentals of saRNA functions, specific mechanisms underlying the activation of cytosolic sensors in the innate immune system, and saRNA adjuvant and cytotoxic effects, as well as approaches to decrease reactogenicity and improve saRNA efficacy through optimization of saRNA sequence.

In the first part of our review, we analyze a number of preclinical and clinical studies, which demonstrate the potential of saRNA for vaccination purposes. The second part of our review systematizes current data on the influence of non-structural proteins of alphaviruses of various origins on cell homeostasis and the innate immune response. We analyze various currently used approaches to optimize saRNA products that are aimed at reducing cytotoxicity, enhancing replication efficiency, and increasing the expression of the gene of interest. In the third part of this paper, we describe the fundamental causes of saRNA reactogenicity. Understanding these mechanisms may contribute to the development of more effective and less reactogenic saRNA-based therapeutics.

## 2. Safety of saRNA-Based Therapeutics

Although the saRNA platform shows promise and saRNA-based products have already been approved for clinical use (ARCT-154), concerns regarding its safety still stand. In preclinical studies on rodents, mild-to-moderate local side effects (erythema and/or edema) were registered at the injection site of saRNA-based therapeutics (Table 1). Furthermore, laboratory rodents exhibited systemic effects such as changes in hematological parameters (increased levels of neutrophils and divergent levels of monocytes and lymphocytes) and increased expression levels of pro-inflammatory cytokines and chemokines. Notably, in three studies from the Rockville Center for Vaccines Research, elevated AST and ALT levels (markers of hepatotoxicity) were registered in female rodents [15,22,23]. The negative effects observed in rodents might arise from high doses of saRNA-based therapeutics per body mass (12–15 μg). However, elevated ALT and AST levels were also observed in primates of the *Cercopithecidae* family. A study on *Macaca fascicularis* [24] showed that administering 100 μg of the molecular adjuvant (IL-12) encoded in saRNA increased the plasma levels of the inflammatory markers (increased content of fibrinogen, products of its degradation, and C-reactive protein) in males, as well as elevating ALT and AST levels in females. While elevated ALT and AST levels are a classic marker of hepatotoxicity, they can also indicate muscle injuries [25]. Increased ALT levels can be associated with cell damage in liver tissues [26,27]. Elevated AST and ALT levels in experimental animals might be related to administration of extremely high doses of the therapeutics. For instance, a clinical study of the COVID-19 saRNA vaccine showed that the maximum tolerated dose of ARCT-021 was 7.5 μg [28], which was significantly lower than in preclinical studies. The sensitivity of females to saRNA may be related to varying levels of sex steroid hormones, which can affect the intensity of the innate immune response, as well as humoral and cell responses [29].

Preclinical trials of mRNA therapeutics based on linear RNA, such as RNA-1273 and BNT162b2, demonstrated elevated cytokine blood levels; increased counts of leukocytes, neutrophils, and eosinophils; decreased lymphocyte numbers; elevated levels of AST, alkaline phosphatase, triglycerides, cholesterol, and bilirubin; and reduced total protein content [31,32]. Therefore, the results of preliminary clinical studies demonstrated the potential for adverse effects, which manifested as both local and systemic reactions in response to the administration of high doses of the saRNA. It was also evident that the judicious selection of dosage in clinical trials on humans was imperative, given the direct correlation between the incidence of adverse effects and dosage.

Results of clinical trials of saRNA products (Table 2) also showed moderate local adverse effects after vaccination, including pain, swelling and erythema at the administration site. Higher doses provoked systemic effects such as fatigue, headache, myalgia, chills/rigors, and arthralgia. Only a small number of participants showed altered hematological (changes in neutrophil numbers) and blood biochemical (elevated ALT and AST) parameters. Clinical trials of the mRNA-based vaccines RNA-1273, BNT162b2, and CS-2034 [33,34,35] also demonstrated dose-dependent local and systemic adverse effects, including inflammation at the administration site, fatigue, headache, chills, muscle and joint pain, and altered hematological and blood biochemical parameters.

Some clinical trials employed the VEEV TC-83 strain carrying the G3A mutation. This reduced excessive subgenomic RNA transcription and increased IFN-I production, likely enhancing the self-adjuvanticity of the vaccine. However, this modification increased the risk of reactogenicity. In the NCT04934111 clinical study, the authors introduced the sequence of the innate immune response inhibitor ORF4a from MERS-CoV into the cassette containing the transgene [40]. Such modification, most likely, had the opposite effect on the induction of IFN-I expression when compared to the G3A mutation. In clinical studies, the dose of saRNA did not exceed 10 μg and was sufficient to elicit the adaptive and protective immune responses. Overall, the data from clinical studies of saRNA-based vaccines indicate that they have comparable levels of reactogenicity to classical mRNA vaccines at low doses; however, saRNA exhibits a more pronounced dose-dependent increase in reactogenicity.

It is important to note that the adverse effects associated with the use of RNA vaccines may also be due to the LNP delivery system [42,43]. It is noteworthy that lipid components, including SM102, ALC-0159 and PEG2000-DMG, have been observed to demonstrate toxicity at elevated dosages. However, the doses employed in saRNA vaccines are considerably lower and are anticipated to contribute to a lesser extent to the toxic effects [31,44]. Consequently, the contribution of lipids to the reactogenicity of saRNA does not appear to be pivotal. It is imperative to modify biological characteristics of saRNA to improve its safety.

## 3. Optimization of Non-Structural Proteins to Improve Safety of saRNA Products

One approach to increasing the safety and efficacy of saRNA-based therapeutics involves optimizing the RNA sequence that encodes nsPs proteins, which carry out the self-amplification process. It is hypothesized that alterations in the sequence of RNA encoding nsPs may have the potential to reduce reactogenicity and enhance the translational efficiency of saRNA. In the context of vaccine development, it is imperative to achieve a balanced immunostimulatory effect, a moderate inflammatory response, high efficiency, and long-lasting translation of target genes. Conversely, the development of gene therapy drugs is predicated on the attainment of three fundamental objectives: the mitigation of immune stimulation, the maintenance of target protein expression, and the absence of genotoxicity.

### 3.1. saRNA Replication

**Figure 1:**
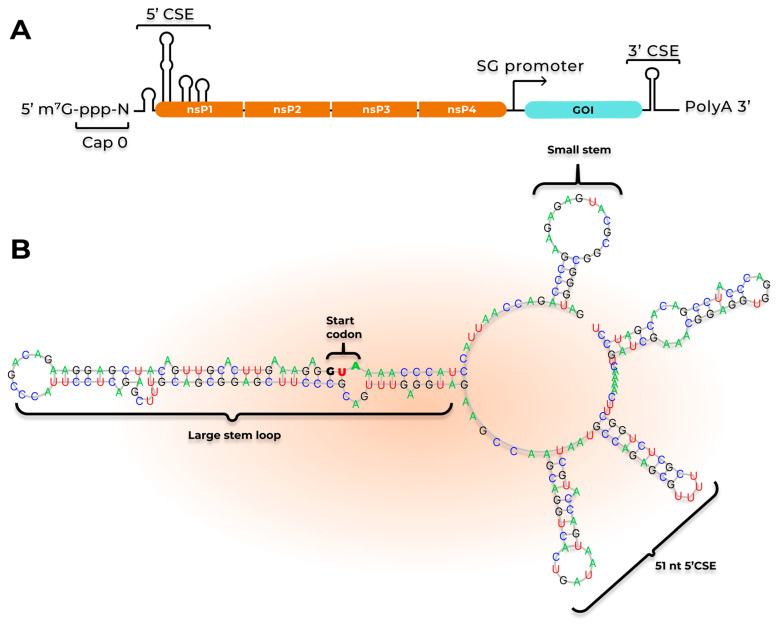
Structural and functional characteristics of saRNA. (**A**) saRNA structure: 5′-Cap 0 (N7mGppp); 5′-CSE (conserved sequence element)—includes 4 stem-loop structures, functionally facilitating the recruitment of the ribosome complex and translation factors, as well as the synthesis of (+)RNA by RdRp; nsP1–4—region encoding non-structural proteins; SG (subgenomic) promoter—functions as a ribosome binding site and as a start site for the synthesis of subgenomic (+)RNA; GOI (Gene of Interest)—sequence encoding target proteins; 3′-CSE—synthesis of (−)RNA by RdRp; Poly(A) tail—potential binding site for PABP proteins and formation of the translation initiation complex. (**B**) Structure of the 5′-CSE region. Includes 4 stem-loop structures involved in translation initiation and (+)RNA synthesis.

The saRNA sequence contains the elements of alphavirus genomic RNA, except for structural genes, which are replaced with target sequences (genes of interest, GOIs). saRNA is a sense RNA that ranges in length from 8 to 15 kb depending on the GOI, featuring a 5′ cap structure, regulatory sequences known as conserved sequence elements (CSE), non-structural genes that encode proteins required for saRNA replication, a subgenomic promoter (SG), and a poly(A) tail (Figure 1). The 5′ cap is a Cap 0 structure (N7mGppp), which, similar to viral RNA, allows saRNA to mimic host cell mRNA; however, the lack of methylation at the ribose 2′-O positions in the first two nucleotides may lead to an increased likelihood of stimulating innate immune receptors [45]. A distinct feature of the 5′ end of saRNA is that the sequence begins with an AU motif, immediately followed by a motif that forms a stem-loop structure required for the RNA-dependent RNA polymerase (RdRp) [46]. Changes in this region lead to either a reduction or a complete loss of the ability of saRNA to replicate [46]. Similar to mRNA, saRNA contains a poly(A) tail at the 3′ end [45]. According to the model proposed by Frolov et al., the 5′ cap and poly(A) tail likely function similarly to eukaryotic mRNA: they spatially converge and interact with cellular factors, which initiate viral RNA translation [47]. The critical regions for saRNA replication, the 5′-CSE and 3′-CSE, form the stem-loop structures required for recognition by the RdRp complex (Figure 1A). Figure 1B presents a possible secondary structure of the first 230 nucleotides of saRNA based on the genome of the *Venezuelan Equine Encephalitis virus* (VEEV). Michel et al. demonstrated that mutations in the 51-nucleotide region of the VEEV genome significantly reduced saRNA replication activity [48]. This sequence is located at the 5′ end of saRNA, contains two stem-loop secondary structures, and encompasses part of the sequence encoding the nsP1 protein (Figure 1B). Additionally, the small stem and large stem-loops presented in Figure 1B play a significant role both in the replication and translation of non-structural genes [46,48]. These data indicate that these motifs are critical for proper saRNA function.

**Figure 2:**
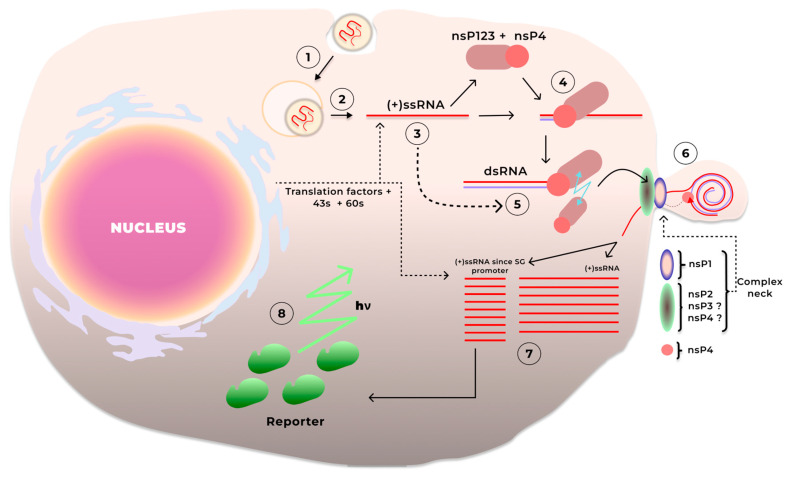
saRNA functions in the cell: 1—Endocytosis of lipid nanoparticles containing saRNA. 2—saRNA is released from endosomes and exit into the cytoplasm. 3—Recruitment of translation factors and ribosomes, and translation of non-structural proteins nsP123 and nsP4 (or nsP1234, followed by cis-cleavage of the polyprotein into nsP123 and nsP4). 4—Synthesis of antisense RNA by the primary complex nsP123 and nsP4 in the cytoplasm (synthesis direction relative to the template strand from 3′ to 5′). 5—After the accumulation of nsP123 and nsP4 proteins from step 3, nsP123 is first trans-cleaved into nsP1 and nsP23, then nsP23 is cleaved into nsP2 and nsP3. 6—Formation of spherules through the action of nsP1, as well as nsP2 and nsP3 proteins; sequestration of dsRNA from the cell cytosolic compartment, sense RNA synthesis in spherules using nsP4, capping mediated by nsP1, and (+)RNA release into the cytosol. 7—Two types of sense RNA are synthesized: a full-length saRNA sequence and one truncated sequence containing the SG promoter and poly(A) tail; GOI translation is initiated at the SG promoter. 8—Accumulation of the protein product.

After entering the cell, saRNA recruits the translation initiation factors and ribosomes, initiating the translation of non-structural gene sequences (Figure 2). At the first stage, the non-structural gene sequence is translated into the nsP1234 polyprotein, which undergoes proteolytic cleavage at the junction of proteins nsP3 and nsP4 into two parts, nsP123 and nsP4, through cis-interaction with the region of the nsP2 protein that possesses protease activity [49,50]. This configuration of the replication machinery is active at the early stages of saRNA replication and facilitates the synthesis of antisense RNA, leading to the formation of dsRNA. It is likely that when the concentrations of nsP123 and antisense RNA reach relatively high levels, the nsP2 domain selectively hydrolyzes the nsP123 polyprotein into the individual proteins nsP1, nsP2, and nsP3 through trans-interaction. At this stage, the synthesis of antisense RNA declines, while the synthesis of sense genomic and subgenomic RNA is initiated. This process occurs in membrane invaginations called spherules, formed by the action of the non-structural proteins nsP1, nsP2, nsP3, and nsP4 [51,52,53]. In the spherules, dsRNA and the replication machinery are sequestered from the cytosol, preventing their interaction with pattern recognition receptors (PRRs). The non-structural proteins form pore-like structures between the spherule and the cytosol, known as the neck complex [52], facilitating selective transport between compartments. The synthesis of subgenomic single-stranded (+)ssRNA is initiated at the subgenomic promoter, and it is subsequently used as a template for translating target proteins in the cytosol.

### 3.2. The Influence of Non-Structural Proteins of Alphaviruses on the Cell and the Efficiency of saRNA Replication

Each non-structural protein contributes to saRNA replication. However, apart from their direct role in its replication, non-structural proteins also affect cellular processes such as transcription, translation, and the interferon response. The functional properties of non-structural proteins and their influence on the cell are determined by the origin of viral elements used in the design of the saRNA platform (Table 3).

#### 3.2.1. Arthritogenic Alphavirus Genome-Based saRNA

Arthritogenic viruses include *Sindbis virus* (SINV), *Chikungunya virus* (CHIKV), *Semliki Forest virus* (SFV), and *Ross River virus* (RRV). These representatives of the *Togaviridae* family induce a cytopathic effect (CPE), primarily in joint tissue cells [45]. Using *Chikungunya virus*, we have shown that CPE arises from disruption of the functional integrity of the endoplasmic reticulum due to cell stress and autophagosome formation, ultimately leading to apoptosis approximately 24 h after infection [66]. Cell death induction can occur for several reasons: as a result of the activation of the innate immune response upon viral infection or LNP containing saRNA, and/or due to the specific action of non-structural and structural proteins. We will not discuss the action of structural viral proteins, since they are not encoded by saRNA-based therapeutics. Experimental data indicate that the SINV nsP2 protein rapidly suppresses cellular transcription [60]. The model of transcription termination is as follows: The non-structural protein nsP2 binds to the host cell DNA due to its helicase activity, altering the torsional stress of DNA while simultaneously interacting with the DNA-dependent RNA polymerase II. This results in polymerase stalling, activation of the repair system, and degradation of the catalytic subunit [60]. The degradation of Rpb1, the catalytic subunit of the RNAPII complex, is mediated by its ubiquitination due to polymerase stalling at the binding site of nsP2 with DNA [60]. Other studies, focused on arthritogenic viruses, describe similar effects of the nsP2 protein, confirming a common mechanism of inducing cell death via Rpb1 degradation [66,67]. These results indicate that the use of non-structural proteins of arthritogenic alphaviruses as replication machinery for saRNA design should be approached with caution and accompanied by measures to prevent the undesirable effects of the nsP2 protein. Therefore, when employing saRNA based on the genomes of *arthritogenic alphaviruses*, it is necessary to modify the nsP2 sequence in order to reduce reactogenicity and avoid other undesirable effects.

#### 3.2.2. saRNA Based on the Genomes of Encephalitic Alphaviruses

Encephalitic alphaviruses include Venezuelan Equine Encephalitis Virus (VEEV), Eastern Equine Encephalitis Virus (EEEV), and Western Equine Encephalitis Virus (WEEV). The cytopathic effect induced by the non-structural proteins of encephalitic alphaviruses differs from the mechanism of action of arthritogenic alphavirus proteins. Rather than the nsP2 protein, the main effectors of cytotoxicity in encephalitic alphaviruses are capsid proteins, which block nuclear pores, disrupting mRNA signaling and transport from the nucleus [68,69]. Thus, when using VEEV-based saRNA, one can expect no impact on nuclear transport in the cell, since the saRNA lacks sequences for capsid proteins. This feature is one of the reasons why using the VEEV replication machinery for saRNA design is such an attractive prospect. Although encephalitic alphavirus-based saRNA has no obvious direct impact on transcription in the cell, there are certain mechanisms that reduce saRNA replication activity. Excessive replication of VEEV genome-based saRNA can be limited by superinfection exclusion (SIE), facilitated by the activity of the C-terminal hypervariable domain (HVD) of the nsP3 protein, which binds cellular factors necessary for saRNA replication [62]. It is possible that this effect of nsP3 is sufficient for reducing saRNA replication efficiency. Thus, saRNA-based therapeutic effectiveness may be reduced when a large amount of saRNA or a combination of several saRNAs enters the cell, as one saRNA may inhibit itself or another saRNA. There is also indirect evidence that the nsP2 protein plays an important role in this process, as indicated by mutagenesis studies [70,71]. We can assume that the cysteine protease nsP2, which processes the nsP123 and nsP23 polyproteins with the release of nsP3, is involved in SIE initiation. Thus, the optimization of the nsP3 sequence is widely used to improve the efficiency of the platform for creating saRNA therapeutics based on the genome of encephalitic alphaviruses.

### 3.3. Modifications That Reduce Reactogenicity

The impact of mutations in the nsP2 and nsP3 proteins on cell homeostasis is evident in a reduction in toxicity and a decrease in the replication rate. A comprehensive summary of the observed mutations and their respective effects is provided in Table 4.

#### 3.3.1. Mutations in Non-Structural Proteins of Arthritogenic Alphaviruses Used for the saRNA Platform

Using VEEV-based saRNA, Frolov et al. showed CPE induction to be significantly suppressed by the mutations P726G and P726L in the gene encoding the SINV non-structural protein nsP2 and incorporated into the VEEV RNA replicon under the subgenomic promoter instead of VEEV structural proteins [70,78]. In BHK-21 cells, these mutations did not affect transcription and translation but moderately reduced the replication rate [70,78]. The P726G and P726L mutations affected nsP2 localization in cell compartments, significantly reducing nsP2 levels in the nuclear fraction, which may indicate its nuclear function. Frolov et al. showed that, along with CPE suppression induced by SINV nsP2 by the mutations P726G and P726L, the cytotoxic effect was also alleviated by the mutations H619Q, H643Q, and P683Q in SINV nsP2 [80]. The P683Q mutation in the saRNA nsP2 protein likely reduced the interaction with the RPB1 domain due to decreased nuclear localization [80], which might mitigate the negative impact on transcription. The P683Q mutation also affected the helicase domain function, preventing the degradation of the RPB1 complex. However, this had minimal impact on cell death [80].

The described mutations in CHIKV genome-based saRNA significantly reduced their CPE [77]. Utt et al. [77] investigated the mutations P718G, E116K, and the insertion of five amino acids GEEGS between the amino acid residues 647 and 648 in the nsP2 protein. As in the case of the SINV nsP2 protein, CHIKV nsP2, encoded by its replicon, performs critical functions in saRNA replication and CPE induction. Along with the insertion of GEEGS between the amino acid residues 647 and 648, the P718G mutation resulted in a non-cytotoxic phenotype in BHK-21 and, to a greater extent, in Huh-7, likely impairing the nsP2 helicase and GTPase activities, the functions associated with CPE [77]. Furthermore, the adaptive mutations F391L in the nsP1 protein and I175L in the nsP3 protein, which have arisen during the experiment, ensured prolonged persistence of saRNA in the human cell line Huh7 [77]. These mutations likely affected the regions that facilitated the recruitment of cellular factors [61], leading to stable saRNA replication in cell lines. Using the CHIKV-based saRNA model, Frolov et al. identified variants of saRNA carrying mutations in the nsP2 protein. These mutations affected the secondary structure of the VLoop at the C-terminus, prevented transcription inhibition, and reduced CPE without any impact on the replication rate. Mutations of three consecutive amino acids A674R, T675L, L676E, as well as the A730V mutation, possibly resulted in the loss of functional activity of the nsP2 helicase domain, impaired DNA binding of nsP2, and prevented the inhibition of IFN-I induction. This likely contributes to the stimulation of the innate immune response and, consequently, antigen presentation, as well as the induction of the adaptive immune response [66]. Thus, the identified mutations in non-structural proteins of *arthritogenic alphaviruses* not only alleviate the cytotoxic effect but also demonstrate a subtle regulatory interplay between saRNA replication efficiency and modulation of the immune response. This opens up prospects for the development of new vaccines and gene therapy solutions based on the saRNA platform.

Thus, for arthritogenic alphaviruses, the main target for reducing the cytopathic effect is the nsP2 protein. Mutations in its helicase or protease domains effectively prevent shutoff of cellular transcription and suppress apoptosis, which is crucial for the safety of saRNA-based medicines.

#### 3.3.2. Mutations in Non-Structural Proteins of Encephalitic Alphaviruses Used for the Development of the saRNA Platform

The structural proteins of *encephalitic alphaviruses* play a dominant role in CPE development. However, the non-structural proteins of their replication machinery, used for the saRNA platform design, also contribute to this process. Petrakova et al. demonstrated that transfection of BHK-21 cells with saRNA carrying mutations Q739L and P773S in the nsP2 protein did not induce CPE or inhibit cell growth, unlike control saRNA without mutations [70]. The nsP2 C-terminal domain is thought to play a critical role in its interactions with host proteins during viral replication, since the localization of this domain is directly adjacent to nsP3. Notably, most mutations are localized in the nsP2 C-terminal domain, which may indicate its role in the cytotoxic effects. Furthermore, the L121P mutation in the nsP3 protein decreases the 5′ VEErep/Pac RNA replicon cytotoxicity in Huh-7 cells [70]. According to another study, mutations in the C-terminal regions of nsP1 (G357C, G1569A, A1572C, C1575T) and nsP2 (A3821T, G3892C, T3922C) also resulted in reduced cytotoxic effects [73]. In addition, the point mutation G3A in the 5′-untranslated region (UTR) of the attenuated strain TS-83 VEEV RNA replicon reduced BHK-21 cell death [73]. This mutation has been associated with decreased susceptibility of the saRNA replication machinery to the interferon response. The G3A mutation, as well as the A30C and C24U mutations, has been suggested to affect hairpin formation in the 5′-UTR, leading to simultaneous changes in similar structures in the 3′-UTR of (–)ssRNA. Together, they cause a change in the genomic/subgenomic RNA ratio and upregulate IFN-α/β in vitro, increasing the percentage of CD4^+^ cells in vivo, compared to VEEV-based saRNA without mutations [72]. The cumulative data on the effects of mutations and SIE indicate a possible functional interplay between the nsP2 and nsP3 proteins of the VEEV RNA replicon, which may play a critical role in its ability to induce CPE. The overwhelming majority of mutations listed in Table 4 that reduce cytopathic effects are localized in the nsP3 sequence. This arises from the regulatory role of nsP3 in saRNA replication. nsP3 facilitates saRNA replication by interacting with cellular proteins CD2AP, SH3KBP1, and FXR, which contain SH3 domains, through its hypervariable domain (HVD). It is possible that mutations in the region encoding nsP3 slightly suppress saRNA replication while reducing excessive activation of the innate immune response.

In contrast to arthritogenic alphaviruses, most optimization attempts for encephalitogenic viruses such as VEEV were focused mainly on the nsP3 protein. Modification of its hypervariable domain allows regulation of replication efficiency by avoiding excessive activation of the immune response. Additionally, mutations may help to overcome replication restrictions imposed by the SIE mechanism. The disparities in the functions of nsPs of saRNA proteins in arthritogenic vs. encephalogenic alphaviruses underscore the heterogeneity of approaches employed to enhance the functional efficacy of saRNA. The expansion of knowledge concerning alphavirus biology may contribute to the diversification of saRNA.

#### 3.3.3. Other Modifications Used in the Development of the saRNA Platform

A potential avenue for expanding the scope of saRNA applications might involve the selection of alternative alphaviruses. Alphaviruses are known to have different tropisms for specific tissues: VEEV effectively penetrates neurons and lymphoid tissues, SINV targets muscle and epithelial cells, while CHIKV infects fibroblasts and joint tissues [81,82,83]. It has been recently shown, using saRNA, that the tropism of alphaviruses for tissues also correlates with replication activity and translation of the gene of interest (GOI) in saRNA based on sequences from related viruses [84]. Thus, saRNA based on the *Everglades virus* (EVEV) genome, a relative of VEEV, was primarily expressed in the spleen, reaching expression levels comparable to those of VEEV [84]. Other saRNA variants based on the genomes of *Mosso das Pedras virus* (MDPV) and *Rio Negro virus* (RNV) were expressed in the lymph nodes and spleen, respectively [84]. These data reflect the flexibility of the saRNA platform, since targeted expression in specific tissues can reduce the risk of reactogenicity.

Uridine analogs are commonly utilized in the production of mRNA therapeutics, which ensures reduced activation of the innate immune response and increased expression of target proteins [3]. Widely used uridine analogs include pseudouridine and N1-methylpseudouridine (m1Ψ) [3,85]. The efficiency of mRNA expression modified with these ribonucleotide analogs is attributed to improved RNA recognition by translation factors, as well as enhanced decoding efficiency on the ribosomes [86,87,88]. As practice has shown, the use of pseudouridine and m1Ψ is not feasible when using saRNA based on the genomic sequences of wild-type alphaviruses due to their impact on the secondary structure of RNA and, consequently, on saRNA replication [89,90]. However, the use of 5-methylcytidine (5meC) in the synthesis of saRNA may serve as an alternative to pseudouridine and m1Ψ in the course of developing the saRNA platform. 5meC does not affect saRNA replication significantly and reduces the likelihood of recognition by PRRs, thereby decreasing IFN-α/β production [89]. Azizi et al. demonstrated that VEEV-based saRNA, modified with 5meC, exhibited higher reporter protein expression, as shown by fluorescence microscopy and flow cytometry in HEK-293T cells [89]. Also, the inclusion of 5meC in saRNA reduces the secretion of IP-10 and IFNa2 in PBMS [91], and in other studies increases the immunogenic characteristics of saRNA [91,92,93].

Another approach involves modifications of RdRp (the nsP4 protein) aimed at enhancing the accuracy and specificity of the catalytic center. This may allow for the effective use of nucleoside analogs, such as m1Ψ. Quintana et al. showed that modification of the catalytic center of VEEV nsP4 (GDD464-466ASR) facilitated the replication of saRNA containing m1Ψ. However, the level of reporter protein expression did not exceed those of saRNA modified with 5meC and the control saRNA sample with unmodified nucleoside triphosphates [75].

On the whole, the presented data support the potential for further optimization of the saRNA platform for developing novel therapeutic and preventive solutions. The ability to introduce mutations into the sequences encoding nsP2 and nsP3 proteins and target gene expression in specific tissues by selecting suitable alphavirus genomes, as well as the inhibition of PRR activation by nucleotide analogs, will allow for reducing CPE and elevating the efficiency of target gene expression. However, it should be noted that reducing the cytopathic effect alone is insufficient for fine-tuning the saRNA sequence. In order to optimize the sequence, it is imperative to conduct a thorough study of the interaction between the saRNA and immune system components. A reduction in the proportion of dsRNA, coupled with the modulation of the interaction of nsP with cellular factors, may reduce the likelihood of unwanted inflammation. This reduction is imperative for the success of the saRNA-based therapy.

## 4. saRNA and Innate Immunity

### 4.1. The Role of Innate Immunity Receptors in saRNA Recognition

Understanding the mechanisms underlying the interaction between saRNA and the components of the innate immune system is crucial for developing an effective saRNA-based therapeutic platform.

saRNA processing is a tightly regulated multistep process, which comprises the synthesis of three different types of RNA [45]. First, saRNA directs the synthesis of the full-length (-) strand, which, in turn, serves as a template for the synthesis of the full-length (+) RNA (a copy of saRNA) as well as the transcription of subgenomic RNA translated into the target protein.

Thus, during saRNA replication in the target cell, alongside the (+) strand of subgenomic capped RNA, dsRNA complexes and RNA with a free 5′-triphosphate group are invariably formed. These RNA intermediates can stimulate pattern recognition receptors (PRRs), such as Toll-like receptors (TLRs), NOD-like receptors (NLRs), and RIG-I-like receptors (RLRs) [94].

The key cellular sensors activated in response to the internalization of saRNA are PRRs, which have intracellular localization include the endosomal receptors TLR3, TLR7/TLR8 and the cytosolic sensors RIG-1 and MDA5 [95].

**Figure 3:**
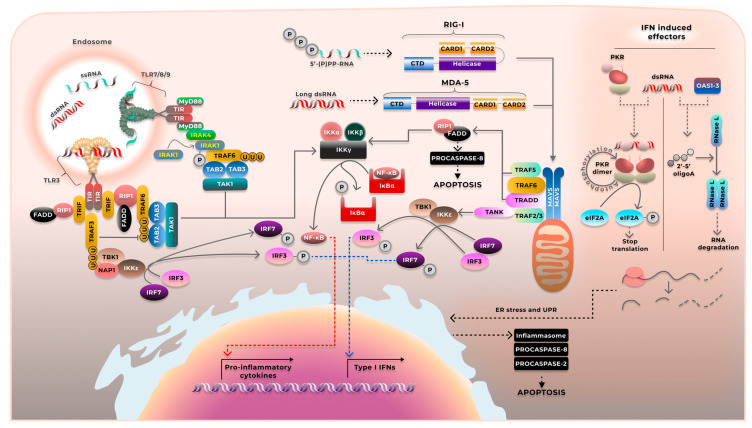
Pathways underlying PRR activation in response to saRNA The intermediate products of saRNA replication are primarily capable of interacting with TLR3, RIG-I, and MDA5. TLR3 receptors are located on the membranes of endosomes and, in some cases, on the outer plasma membrane. TLR3 activation leads to the phosphorylation of tyrosine residues (Y759 and Y858) on the TIR domain of the TLR3 receptor, which triggers the formation of signaling complexes through the adapter proteins TRIF, TRAF6, and TRAF3. TRAF proteins function as ubiquitin ligases and scaffolds. Their activation causes either the release of the p50/p65 complex (NF-κB) and the activation of pro-inflammatory cytokines, or IRF-3 phosphorylation and IFN-I activation. RIG-I and MDA5 activation leads to the assembly of the complex comprising MAVS and adapter proteins on the membranes of mitochondria, peroxisomes, or lysosomes. The ubiquitination of these adapter proteins results in the recruitment of kinases, which initiates pathways similar to those activated by TLR3. IFN-I stimulates the expression as well as the activation of PKR and the OAS family proteins. Upon interacting with dsRNA, PKR transitions to an active state, undergoes dimerization, and trans-phosphorylates PKR dimers, converting them into an active state. PKR phosphorylates eIF2A, preventing the formation of the 43S ribosomal initiation complex, and halting cellular translation. OASs, upon interacting with dsRNA, synthesize 5′-2′-oligoadenylates, which activate RNase L. RNase L undergoes dimerization and hydrolyzes cellular mRNA. All pathways are capable of inducing apoptosis in one way or another.

The precise role of various PRRs in recognizing saRNA and inducing the innate immune response still has to be clarified. The primary PRR-mediated cell signaling pathway activated in response to the introduced saRNA is detailed in Figure 3.

TLRs that recognize saRNA are localized in the endoplasmic reticulum, endosomes, and lysosomes. TLR3 predominantly recognizes dsRNA, while human TLR7 and TLR8 recognize ssRNA [96,97].

While TLR3 and TLR7/8 recognize different forms of RNA, they mediate metabolic pathways via various signaling adapters.

TLR3 activation triggers its interaction with the adapter protein TRIF through the intracellular C-terminal domain (TIR), which promotes signal transduction via two mechanisms. According to the first mechanism, the adapter protein TRAF3 recruits the protein NAP1, which, in turn, recruits the serine/threonine kinases TBK1 and IKKε. Together, they phosphorylate the transcription factor IRF3, leading to the transcription of the interferon-beta (IFN-β) genes. The second mechanism depends on the TLR3 interaction with the adapter proteins TRIF6 and RIP1. TLR3 activates the serine/threonine kinases TAK1, causing NF-κB release. NF-κB translocates to the nucleus and activates the transcription of the proinflammatory cytokines and antiapoptotic proteins (the members of the Bcl2 family and cellular inhibitors of apoptosis) [98,99].

saRNA interaction with TRL7/8 activates the MyD88-dependent pathway. MyD88 stimulates IRF7 via the interaction with IRAK1, IRAK4, IKKα, TRAF3, and TRAF6, rapidly inducing the synthesis of interferons (predominantly IFN-α) and proinflammatory cytokines [97].

Intracellular TLRs are thought to be responsible for the initial recognition of nucleic acids released from the endosomes [95]. However, after RNA/DNA translocates to the cytosol and initiates replication, forming intermediates, the primary role in inducing the innate immune response is mostly carried out by cytosolic sensors such as RIG-I and MDA5.

RIG-I and MDA5 are RNA helicases that bind dsRNA. RIG-I activation is thought to be mediated by dsRNA, which contains di- or triphosphate groups at the 5′-end, while MDA5 is activated by recognizing long dsRNA [100]. The interaction between the ligand and the C-terminal domain of the sensor triggers conformational changes and induces downstream signaling by binding with the mitochondrial antiviral-signaling protein MAVS [101,102]. Further signal transduction is mediated by the adapter proteins TRAF5, TRAF6, and TRAF2/3. It involves the activation of either IRF3 with subsequent IFN-α/β or NF-κB, which induces the transcription of antiapoptotic and proinflammatory genes [103].

Since signaling pathways for various receptors largely overlap and compensate for each other [104], their exact role in recognizing saRNA has not been clarified yet.

The upregulation of genes encoding the innate immune response receptors can serve as indirect evidence of their activation [105]. Studies on the *nsP3* saRNA sequence optimization have shown that 24 h after saRNA administration, it upregulates *TLR3*, *MDA5*, and *RIG-I* in HeLa cells, in contrast to non-replicating capped mRNA [17].

Nikonov et al. showed that SFV-based saRNA replication in murine fibroblasts caused RIG-I and MDA5 activation, inducing IFN-α/β production [106]. MDA5 and RIG-I knockdown significantly reduced saRNA-dependent IFN-β production, while full knockout of these cytosolic receptors did not cause IFN-β stimulation which more prevent effect repression of saRNA replication. Furthermore, RdRp was shown to suppress IFN-β induction. These results imply that activation of the cytosolic sensors relies on the formation of dsRNA intermediates during saRNA amplification.

The reactogenicity of VEEV-based saRNA in C57BL/6 mice was compared to that of C57BL/6 mice with deficient RDR-adapter MAVS or knockout of MyD88/TRIF genes, which are responsible for TLR7 signal transduction. Blood IFN-α and IFN-β levels in MAVS(–) mice were significantly decreased in comparison to other groups [104].

Akhrymuk et al. studied the innate immune response against VEEV and SINV and showed that two cytosolic sensors, RIG-I and MDA5, played equally important roles in inducing the primary anti-alpha viral response during the first hours post infection [107]. According to that study, sequestered saRNA intermediates, including dsRNA in spherules, failed to recognize TLR3, TLR7, and TLR8.

In summary, along with TLR3, TLR7, and TLR8 activation by saRNA, RIG-I and MDA5 are also essential for the response to saRNA in the presence of residual dsRNA, uncapped RNA, and unmodified residues in IVT preparations. At the same time, RIG-I and MDA5 play a major role in recognizing replicating saRNA and determining the activation of protective mechanisms, stimulating IFN-I production and inducing proapoptotic signaling.

### 4.2. Innate Immunity Components Activated in Response to saRNA

Negative consequences of the functional interaction between saRNA and the innate immune response receptors include the inhibition of transgene transcription and translation as well as the induction of apoptosis.

Transgene transcription inhibition is mediated mostly by IFN production and activation of a number of IFN-stimulated genes (ISGs). Therefore, the activation of the innate immune response receptors triggers the signaling pathways mediated by the transcription factors NF-κB and IRF3, leading to the stable expression of IFN-I genes. IFN-I stimulates the expression of the IFN-stimulated genes (ISG), such as protein kinase R (PKR), 2′-5′-oligoadenylate synthase (OAS), RNase L, and IFN-induced proteins with tetratricopeptide repeats (IFIT) [108,109]. ISGs can directly hinder saRNA replication and expression.

PKR contains a domain that directly binds dsRNA, including secondary RNA structures (hairpins) that imitate dsRNA [110,111]. After dsRNA activation, PKR undergoes dimerization and autophosphorization and, in turn, phosphorylates initiation factor eIF2α. eIF2α phosphorylation halts translation of both cellular mRNA and IVT RNA [112,113].

PKR activation after oxidative stress has been shown to sensitize cells to apoptosis. Prolonged PKR activation induces apoptosis via the proapoptotic factor FADD with the subsequent activation of caspase 8. Furthermore, PKR activation leads to BAK and BAX protein expression and induces apoptosis via the mitochondrial mechanism and cytochrome C release [114,115].

Along with PKR, the PRK-like endoplasmic reticulum kinase (PERK) may play a significant role in the negative regulation of translation by alphaviruses. In particular, PERK performs a dual function upon VEEV, playing a notable role in viral replication [116]. On the one hand, PERK may be required for the translation of the non-structured alphavirus proteins at the early stages of infection. On the other hand, at later stages, PERK inactivation causes cell death. Nevertheless, it should be noted that PERK activation can be induced by ER stress, caused by the production of alphavirus proteins, including misfolded proteins [117]. Thus, the role of PERK in saRNA-induced apoptosis is still to be clarified.

The *OAS* gene family (2′-5′-oligoadenylate synthases) is a group of genes ubiquitously expressed in mammals that play an important role in the antiviral response. This family includes four genes: *OAS1*, *OAS2*, *OAS3* and *OASL*. OAS acts as sensors of viral infection, directly recognizing dsRNA and triggering the synthesis of 2′-5′-oligoadenylates (2-5A) [118]. The OAS1, OAS2, and OAS3 proteins interact with RNase L with different affinity, causing RNAse activation [119]. The fourth gene encodes the OASL polyprotein, which promotes RIG-I signaling without RNase L activation [120].

Human RNase L is an enzyme with latent activity expressed in most cells and tissues. It is a unique endoribonuclease with very low RNA sequence specificity and is regulated by complex mechanisms [121,122]. RNase L comprises the N-terminal regulatory domain containing ankyrin repeats, the protein kinase homology domain, and the C-terminal ribonuclease domain. RNase L is an effector of the 2-5A system. Its regulatory domain interacts with 2-5A inducing conformational changes, which, according to current data, expose the C-terminal ribonuclease domain. This allows RNase L to form homodimers and gain the nuclease activity [123]. Signaling regulation is mediated by 2′-phosphodiesterase and 5′-phosphatase, which apparently induces transient RNase L activation only in the presence of high dsRNA content [124,125,126]. RNase L activation by exogenous 2-5A leads to degradation of 70% of cellular RNA.

There were studies focused on the changes in OAS1 and OAS3 expression in response to saRNA based on the wild-type VEEV genome (wt saRNA) and saRNA harboring mutations in the sequence of the *nsP3* gene (Q48P or I113F) and linear non-replicating mRNA. Using qPCR, both (wild-type as well as mutated) saRNAs were shown to significantly upregulate OAS1 and OAS3 24 h after transfection in HeLa cells [17]. 28S/18S fractions of ribosomal RNA (rRNA) isolated from HeLa cells at various time points after transfection with saRNA were analyzed by capillary electrophoresis [17]. Wild-type saRNA was shown to cause significant rRNA degradation after 24 and 48 h, which was not the case for mutated saRNA. This implies that rRNA degradation takes place after transfection with saRNA, which likely relies on the activation of the OAS-RNase L pathway and depends on mutations in the non-structured proteins of alphaviruses.

The activity of caspases 3 and 8 was estimated by Western blotting, while apoptosis was assessed by dual staining with annexin V and propidium iodide [17]. Gong et al. demonstrated that mutant saRNA caused lower apoptosis levels compared to wt saRNA, which resulted in higher expression of the target protein.

Takata et al. showed that at least three ISGs, including ZAP, IFIT1, and IFIT3, significantly affected VEEV replication, which was also relevant for the saRNA derived from the genome of this alphavirus. ZAP recognizes CG dinucleotides present in alphavirus RNA [127], while IFIT1 and IFIT3 bind the 5′-termini of viral Cap0-RNA inhibiting alphavirus translation [108,128].

In contrast to conventional linear mRNA, a strong innate immune response to saRNA takes place at later stages of RNA amplification. Current data indicate that modification of saRNA-based alphavirus components allows for the generation of saRNA with reduced immunogenicity and stable transgene expression [17] 17. However, the replication ability of saRNA raises reasonable concerns regarding the biosafety of this platform, since it can induce cytotoxicity and immune-related adverse effects.

### 4.3. Alleviating saRNA Immunogenicity by Regulating Intracellular Interactions

Researchers initially examined the combined use of immunosuppressive therapeutics to improve the efficiency of IVT RNA transfection in regenerative medicine studies [129]. The enhanced translation of an mRNA-encoded transgene was achieved using the steroid anti-inflammatory therapeutic dexamethasone, which apparently relied on it inhibiting the NF-κB pathway [130].

Employing the innate inhibiting proteins (IIP) for selective inhibition of the innate immunity pathways is another strategy for reducing the excessive immunogenicity of saRNA [131]. Certain viruses have developed mechanisms to avoid the activation of the IFN-1 system by a host cell, which provides them reproductive advantages. One of the promising approaches is utilizing viral proteins as antagonists of the IFN-I system to alleviate innate immune responses to saRNA.

The combination of the vaccinia proteins E3, K3, and B18R (ERB complex) with the non-structural influenza virus protein NS1 has been shown to increase the translation efficiency of IVT RNA both in vitro and in vivo [132,133]. These proteins utilize different mechanisms for the stimulation of transgene (luciferase) translation and inhibition of mRNA-dependent apoptosis. E3 and K3 inhibit PKR activation, whereas B18R disrupts IFN-I signaling, preventing the interaction between extracellular IFN and the IFN-α/β receptor (IFNAR) on the cell surface [134]. NS1 protein exerts multiple immunosuppressive functions by inhibiting key ISGs, such as PKR, OAS, IRF3, and NF-κB, as well as interacting with the nuclear factor CPSF30, which, in turn, impairs pre-mRNA processing in the nucleus and leads to global translation inhibition of cellular proteins [135,136].

Blakney et al. studied the influence of IIP on translation of the saRNA-encoded transgene using 10 viral proteins that suppress the innate immune response: influenza A NS1 protein, herpes simplex virus type 2 (HSV-2) US2 protein, herpes simplex virus type 1 (HSV-1) US1 and US2 proteins, *farmyard pox* (ORF) OV20 protein, *bovine viral diarrhea* virus Npro protein, parainfluenza virus type 5 V-protein, MERS-CoV M-protein and ORF4a protein, flavivirus *Langat* NS5-protein [137]. They designed 10 constructs encoding the VEEV-based saRNA regulated by the subgenomic promoter, which produced one of the abovementioned proteins separated from the *fLuc* reporter gene by the protease cleavage T2A site. *fLuc* luminescence intensity in HEK293T.17, HeLa, and MRC5 cells after saRNA delivery using the polymeric pABOL system, which is resistant to the innate immune response due to its bioreducible properties [138].

MERS-CoV V, PIV-5 and ORF4a proteins were shown to upregulate transgene expression by 100–500 times in vitro in the IFN-sensitive HeLa and MRC5 cell lines. Both proteins prevented IRF3 and NF-κB activation compared wt saRNA (without IIP) resulting in the reduced IFN-I and ISG production, which mitigated the excessive response to the high dose of saRNA [137].

Wojcechowskyj et al. demonstrated that the leader peptide from *Cardiovirus* encoded by mRNA (called RNAx) alleviated excessive PRR activation and promoted target gene expression [30]. RNAx prevented PRK-mediated translation inhibition by interfering with nuclear–cytoplasmic transport. Using the murine C57BL/6 model, they showed that the combined administration of 2 μg of saRNA encoding NanoLuc and mRNA-RNAx led to a statistically significant 170-fold NanoLuc upregulation compared to the group receiving only saRNA.

Another promising tool for evading the innate immune response is the leader protein of Theiler’s murine encephalomyelitis virus (TMEV L) belonging to the *Cardiovirus* genus as well [30,139]. Immunization of C57BL/6 mice with the vaccine, based on the VEEV-derived saRNA encoding TMEV L, reduced production of the cytokines IFN-α, IL-6, KC, MCP-1 and TNF-α both upon initial administration and boosting. Meanwhile, it did not affect the Th1/Th2 cell response and elevated the levels of neutralizing antibodies [30].

One of the alternative approaches for reducing PKR activity upon administration of SINV-based saRNA is the expression of the mutant PKR (K296R) under the SG promoter. Fusing saRNA with cis-encoded mutant PKR drastically upregulated GFP in HEK293T, HeLa, and A549 cells compared to non-fused saRNA [79]. Introducing additional mutations into the nsP2 protein in the saRNA-encoding construct promoted 3–4 times higher eGFP expression.

In conclusion, we emphasize that achieving optimal immunogenicity requires a careful balance between the innate immune response and transgene expression. Although alphaviral replicons possess adjuvant properties, they can inhibit both T cell [140,141] and B cell [137,140,142,143] immune responses due to the excessive activation of the IFN-I signaling. A decline in the protective efficacy of 13% of participants in the clinical trials of saRNA-based vaccines can be explained to the overactivation of the innate immune response and the suppression of transgene expression encoded by the saRNA [36,144]. These immunological aspects should be taken into account while developing saRNA-based therapeutics. As the pathways of innate immunity activation described herein create significant barriers to the efficient expression of transgenes, the optimization strategies described in Section 2 may be applied to reduce the negative impact of their activation. To date, a number of approved technological solutions have made it possible to significantly improve saRNA functional efficacy.

## 5. Conclusions

The saRNA platform has a series of advantages when compared to classic linear mRNA. Despite certain concerns regarding increased reactogenicity, more and more solutions to this problem are emerging. The approval of the first saRNA-based vaccine in 2024 and other clinical studies indicate the high potential of saRNA-based therapeutics. However, researchers continue to optimize the saRNA sequence to reduce the reactogenicity and to increase the translation efficiency of target proteins. It should be noted that all existing saRNA therapeutics with published clinical trial results are VEEV-based. A key player in the immune response to such encephalitic alphavirus-based saRNA is the nsP3 protein, which plays an important role in regulating saRNA replication and dsRNA accumulation, resulting in modulation of the immune response. Mutations in non-structural proteins solve the problem of saRNA reactogenicity to a certain extent. The use of saRNA with a mutated nsP4 gene may also allow the use of nucleoside analogues, including uridine. Prolonged activation of PRRs can trigger IFN-I production, which in turn activates effector molecules such as PKR and RNase L. This cascade may result in local inflammatory reactions and reduce the effectiveness of the saRNA platform. Further optimization of viral sequences and the production of hybrid saRNA variants may allow for obtaining more effective saRNA products.

The degree of self-adjuvanticity can potentially be fine-tuned by introducing mutations, for example, in the nsP3 protein (such as Q48P, I113F, and L121P), which reduce the replication intensity, thereby decreasing the likelihood of deleterious immune responses in host cells. Moreover, mutations in the saRNA sequence combined with the use of nucleotide analogs and inhibitors of innate immune responses could further enhance saRNA effectiveness.

As previously noted, the innate immune receptors RIG-I and MDA-5 play a critical role in limiting saRNA efficacy. Targeted suppression or desensitization of these receptors could thus improve therapeutic outcomes. The application of innate immune response inhibitors such as the Influenza A virus NS1 protein [145], HSV-1 US11 protein [146], Paramyxovirus V protein [147], and MERS-CoV ORF4a protein [148], which interfere directly with RIG-I and MDA-5 signaling by various mechanisms, presents a promising approach to enhance the saRNA platform. This strategy has been recently validated in clinical studies, where saRNA constructs including the MERS-CoV ORF4a sequence exhibited increased translation of the target protein [40,137]. Although the safety of saRNA products requires further assessment, there is already a substantial body of preclinical and clinical trials investigating saRNA-based therapeutics. To date, the majority of studies have focused on vaccine development and cancer immunoadjuvants.

While the application of saRNA in gene therapy remains challenging, incorporating the aforementioned modifications may sufficiently mitigate the innate immune response to saRNA. Thus, saRNA holds significant promise as a versatile platform: on the one hand, it can possess intrinsic self-adjuvant properties suitable for use as an immunogenic vaccine carrier; on the other hand, with appropriate modifications, it may be possible to develop nearly non-immunogenic formulations suitable for gene therapy, leveraging its prolonged self-amplification capability.

In conclusion, self-amplifying RNA as a basis for drug design may become a highly customizable technology platform. As this review shows, the supposed weakness of saRNA—its potent activation of innate immunity—may find applications in vaccine design through rational design. The central theme of current saRNA research is no longer suppression but precise modulation of reactogenicity. Therapeutics based on saRNA could be designed as a potent self-adjuvant vaccine capable of inducing strong and durable immune responses at low doses, as demonstrated by the approved ARCT-154 vaccine. Further development of this platform could include the integration of conditional regulatory systems (e.g., riboswitches) to enhance the controllability of saRNA-based therapies. The development of such regulatory sequences can provide self-amplifying RNAs with the ability to be switched on or off at the right time, thereby reducing the risk of uncontrolled expression of encoded genes. These features, as well as the possibility of using various modifications, make saRNA one of the most flexible and promising platforms for the next generation of RNA therapeutics.

## Figures and Tables

**Table 1:** saRNA safety in animal preclinical trials.

Pathogen/Target	Replicon	Antigen	Dose/Regimen/Tissue	Delivery	Animals	Results and Safety	Reference
Rabies lyssavirus	VEEV	Rabies glycoprotein G	15 μg; 1, 15, 29, 43 days; Blood collection day: 2, 8, 44, 50, 70	Cationic nanoemulsion	Sprague Dawley rats (15f + 15m)	Local effects: mild erythema and/or edema 24 and 48 h after injections. Females: 2nd and 44th days—↑ ALT, ↑ AST, ↓ lymphocytes, ↑ neutrophils, ↑ fibrinogen; 44th day—↑ WBCs. Males: 2nd and 44th days—↓ lymphocytes, ↑ neutrophils, ↑ fibrinogen (+8th day); 44th day—↑ WBCs, ↑ monocytes	[22]
SARS-CoV-2	VEEV	Glycoprotein S	12 μg; 1, 15, 29 days; Blood collection day: 2, 8, 30, 36, 57	LNP	Sprague Dawley rats (15f + 15m)	Local effects: mild erythema 72 h after vaccination. Swelling after 2nd and 3rd doses Females: 2nd day—↓ monocytes; 2nd and 30th days—↑ AST, ↓ lymphocytes, ↑ neutrophils, ↑ fibrinogen; Males: 2nd day—↓ monocytes, ↓ lymphocytes, ↑ neutrophils, ↑ fibrinogen; 30th day—↑WBCs, ↓ lymphocytes, ↑ neutrophils, ↑ fibrinogen	[15]
Rabies lyssavirus and SARS-CoV-2	VEEV	Rabies glycoprotein G and glycoprotein S	12 μg on the 1st day and 15 μg on the 15th day; Blood collection: 4 h, 1 day, 7 day	LNP	Sprague Dawley rats (12f + 12m)	↑ TNF-α, ↑ IL-6—4 h and 24 h after vaccination; Mild erythema in all groups; Female: ↑ ALT, ↑ AST; No signs of toxicity were detected microscopically in the liver	[23]
Cancer	VEEV	Il-12	100 μg, 1, 8, 15 days; Blood collection day: –1, 2, 8, 9, 22	LNP	Macaca fascicularis (1f + 1m)	In the male macaque group, ↑ fibrinogen, ↑ products of its breakdown in plasma, ↑ C-reactive protein were observed after the second injection. On day 9, the female showed a slight increase in ALT and AST levels.	[24]
Influenza	VEEV	Hemagglutinin	0.5 µg 1st and 28th days; Blood collection day: 6 h, 28 days 6 h	LNP	C57BL/6 (n = 5)	↑ IFNα2, ↑ IFNγ, ↑ TNF-α, ↑ KC, ↑ IL-10, ↑ IL-6, ↑ MCP-1 after the first dose; ↑ IFNα2, ↑ IFNγ, ↑ TNF-α, ↑ KC, ↑ IL-10, ↑ IL-6, ↑ MCP-1 after the second dose;	[30]

“↑”: increase, “↓”: decrease.

**Table 2:** Clinical trials of saRNA-based therapeutics.

Name/Phase/Clinical Trial ID	Replicon/Modification/Antigen	Dose	Delivery	Results	Safety	Source
COVAC1 Phase I (ISRCTN17072692)	VEEV/G3A/Glycoprotein S	0.1 μg, n = 39; 0.3 μg, n = 39; 1.0 μg, n = 42; 2.5 μg, n = 24; 5.0 μg, n = 24; 10,0 μg, n = 24; All: n = 192; 1st and 28th days	LNP	Mild ↑ IgG; 6 week—(Nab: 15–43%) 8 week—(SC: 8–57%; Nab: 8 week—11–52%)	Primer and booster 5 μg and 10 μg—100% systemic reaction; The frequency and severity of adverse reactions increased with the dose and also after the booster. Higher grades of headache were more common after the second dose.	[36]
COVAC1 Phase IIa (ISRCTN17072692)	VEEV/G3A/Glycoprotein S	1st day—1 μg; 14th week—10 μg; n = 216	LNP	After 2nd immunization ↑IgG; After 2nd immunization—SC had 80% participants and 56% had NAbs.	Primer 1 μg: Local reaction—53% (soreness/discomfort—49%; pain—20%, erythema—3%; swelling < 1%). No grade 3 (severe) reactions. Systemic reaction—58% (fatigue—29% and headache—29%). Booster 10 μg: Local reaction—94% (pain/discomfort—90%; pain—69%; swelling—2%; erythema—2%). 2 cases—grade 3 local reaction (pain, discomfort). Systemic reactions—88% (fatigue—74%; headache—67%; myalgia—63%; chills/rigors—60%; arthralgia—40%; nausea—27%; fever (≥38 °C)—13%). 24 cases—grade 3 systemic reaction. Neutrophil increase: 11%—not considered clinically significant.	[37]
ARCT-154 Approved (NCT05012943)	VEEV/G3A in 5′-UTR and Q739L in nsP2(US20230256083)/Glycoprotein S	5 μg on the 1st and 28th days. 1, 2, 3a phases: n = 1001; 3b phase: n = 16,107	LNP	4 weeks after the 1st immunization SC—53.8%; 4 weeks after the 2nd immunization SC—94.1%	Prime and booster—myalgia, headache and chills, which resolved quicker than local reactions.	[38]
RBI-4000 Phase I (NCT06048770)	VEEV/G3A in 5′-UTR and Q739L in nsP2(WO2022226019A1)/Rabies glycoprotein G	0.1 μg or 1 μg or 10 μg on the 1st or 57th day. n = 76	LNP	NAb: 67–94% A dose-dependent increase in the titer from 0.1, 1, and 10 μg doses after a booster	Mild local reactions (pain/swelling at the injection site) were observed after single immunization. General reactions: headache and fatigue were the most common (in human studies). The booster dose (second immunization) was found to be more tolerable than the primer dose, with fewer participants reporting adverse effects.	[39]
LNP-nCOV saRNA-02 Phase I (NCT04934111)	VEEV/ORF4a/Glycoprotein S	from 0.1 µg to 10.0 µg on the 1st and 29th day. n = 42	LNP	↑IgG; NAb—91.6% participants at 14 days after stimulation. *No placebo group, imbalance in groups*	Primer: fatigue/malaise (47.6%), headache (42.9%), chills/rigors (40.1%); Booster: fatigue/malaise (63.4%), headache (61.0%), chills/rigors (58.5%); Local reactions—grade 1 and 2, pain (71.4%) and soreness (66.7%)	[40]
ARCT-021 Phase I/II (NCT04668339)	VEEV/-/Glycoprotein S	1 µg, n = 5; 5 µg, n = 10; 7.5 µg, n = 5 (young); 7.5 µg, n = 5 (old); 10 µg, n = 5.	LNP	SC: 80–100%. 5.0 μg and 7.5 μg: best IgG	10 µg dose—many local and systemic suspected AEs, including grade 3. 7.5 µg—mild and moderate reactions (pain, tenderness); The dose-related trend for ≥grade 2 lymphopenia with 0.0%, 25.0%, 26.5%, 30.0%, and 40.0% of participants immunized with 1.0, 5.0, 7.5, and 10 μg doses, respectively. ↑ ALT (5 participants) and ↑ AST (3 participants).	[28]
		3 µg, n = 12 (young); 5 µg, n = 12 (young); 3 µg, n = 12 (old); 5 µg, n = 12 (old); on the 1st and 28th day				
GEMCOVAC-OM Phase III (CTRI/2022/10/046475)	VEEV/G3A/Glycoprotein S	10 μg, n = 2980	LNP	90th day—Nab level: saRNA (754.0) > mRNA (383.1); The 1.76-fold increase in neutralizing antibody titers against Omicron BA.1;	The safety and tolerability of the saRNA vaccine were comparable to those of the mRNA vaccines. Most AEs (adverse effects) were mild to moderate and resolved spontaneously. No vaccine-related serious AEs or deaths were reported. Since mRNA vaccines have been associated with myocarditis, this AE has been included in the study as an AE of special interest; Adult women had a higher immune response than men. Women had significantly more severe local and systemic AEs	[41]

“↑”: increase; SC—seroconversion; Nab—neutralizing anti-S antibody.

**Table 3:** Functional properties of non-structural saRNA proteins.

Protein	Origin	Activity	Functions	Characteristic Function
nsP1	Arthritogenic viruses	Guanine-7N-methyltransferase; guanylyltransferase, scaffold	Catalytic capping [54,55]; membrane anchoring, neck complex assembly, and spherule formation [51,52]	—
	Encephalitogenic viruses			—
nsP2	Arthritogenic viruses	γ, β-ATPase, GTPase, Helicase, 7cysteine protease	Proteolytic cleavage of the nsP1234 polyprotein [49,56]; involvement in RNA capping [55]; helicase activity during replication [57,58,59]	Binding to DNA in the nucleus and RPB1 degradation. Cellular transcription termination [60]
	Encephalitogenic viruses			—
nsP3	Arthritogenic viruses	Homotypic interactions with cellular proteins, regulatory activity	The hypervariable domain (HVD) interacts with FXR cellular proteins, as well as proteins containing the SH3 domain: CD2AP and SH3KBP1, which facilitates replication [61]. The superinfection exclusion (SIE) effect—the limitation of excessive replication through the binding of cellular factors and their depletion in the cell [62]	—
	Encephalitogenic viruses			—
nsP4	Arthritogenic viruses	Rdrp; adenyltransferase activity	RNA synthesis based on an RNA template [63,64]. Polyadenylation of the RNA substrate at the 3′ end [65]	—
	Encephalitogenic viruses			—

**Table 4:** Mutations in non-structural proteins of alphaviruses that alter the effects of saRNA products in in vitro experiments.

Genome	Region	SNV/Insertion	Cell Line	Effect	Origin
VEEV	5′-UTR	(G3A)	BHK-V; L929	Increased genome replication; suppressed subgenomic RNA replication; increased IFN-I levels	[72]
VEEV	5′-UTR	(C24U)	BHK-V; L929	Decreased IFN-I levels	[72]
VEEV	nsP1 and nsP2	nsP1(G357C, G1569A, A1572C, C1575T) + nsP2 (A3821T, G3892C, T3922C)	HEK293T; RAW-Lucia ISG	Reduced innate immune response; increased target protein expression	[73]
VEEV	nsP2	A533I	BHK-V; L929	Reduced cytotoxicity; increased IFN-I levels	[72]
VEEV	nsP2	Q739L, P773S	BHK-21	Possible changes in helicase and protease activities; reduced cytotoxic effects; decreased replication levels	[70]
VEEV	nsP2 and nsP3	G1298R (G3936C) + K1423E (A4311G)	Jurkat; Raw-Lucia ISG; B16F10	Increased replication level; increased overall expression and specific expression of subgenomic RNA; increased IFN-I levels	[71]
VEEV	nsP3	Q48P (A4174C)	BHK-21; Huh7.5.1; C2C12; RAW264.7	Decreased saRNA replication; increased subgenomic RNA translation; elevated innate immune response to saRNA; significantly apoptosis inhibition; increased cell viability compared to wild-type saRNA	[17]
VEEV	nsP3	I113F (A4368T)	BHK-21; Huh7.5.1; C2C12; RAW264.7	Decreased saRNA replication; increased target protein expression; elevated innate immune response to saRNA; significant apoptosis inhibition; increased cell viability	[17]
VEEV	nsP3	L121P	BHK-21	Reduced cytotoxic effects	[70]
VEEV	nsP2 and nsP3	G1298R (G3936C) + S1572G (A4758G)	Jurkat; Raw-Lucia ISG; B16F11	Prolonged reporter expression	[71]
VEEV	nsP3	S1572G (A4758G) + E1584D/ V1634 (G4796T/ G4946A)	Jurkat; Raw-Lucia ISG; B16F12	Regulation of subgenomic (+)RNA translation; reduced subgenomic RNA expression; decreased IFN-I levels	[71]
VEEV	nsP3	E1584/ V1634 (G4796T/ G4946A)	Jurkat; Raw-Lucia ISG; B16F13	Decreased IFN-I levels	[71]
VEEV	nsP3	Sequences 5628–5666 and 5684–5702	BHK-21; Vero	Impaired nsP3-nsP4 proteolytic cleavage	[74]
VEEV	nsP3	Sequence 5628-5666	BHK-21; Vero	Disrupted degradation signaling and cellular localization of nsP3	[74]
VEEV	nsP4	C482Y and K290R	BHK21	Increased accuracy of RdRp; possible use of uridine analogs; influence on replication initiation	[75]
CHIKV	nsP1	A533V	Vero; L929	Reduced cytotoxicity; increased IFN-I levels	[76]
CHIKV	nsP2	A674R T675L L676E A730V	BHK-21; NIH 3T3	Alleviated cytopathic effects due to the changes in the V-loop structure of the nsP2 C-terminus, which inhibits transcription	[66]
CHIKV	nsP2	P718G + GEEGS insert between aa 647 and 648	BHK-21	Reduced cytopathic effects; impaired helicase and GTPase activities	[77]
CHIKV	nsP2	(P718G + GEEGS insert between aa 647 and 648) + F391L + I175L	Huh7	Reduced cytopathic effects; impaired helicase and GTPase activities; prolonged persistence in the cell	[77]
SINV	nsP2	P726L and P726G	BHK-21 NIH 3T3	Reduced cytopathic effects and efficiency of saRNA replication; absent nsP2 nuclear localization	[70,78,79]
SINV	nsP2	H619Q and H643Q. P683Q	BHK-21	Reduced cytopathic effects; absent nsP2 nuclear localization	[80]

## Abbreviations

The following abbreviations are used in this manuscript:

CD2AP	CD2-associated protein
CHIKV	Chikungunya virus
CPE	Cytopathic Effect
CSE	Conserved sequence elements
dsRNA	Double-stranded RNA
EEEV	Eastern Equine Encephalitis virus
FDA	Food and Drug Administration
FXR	Farnesoid X Receptor
GOI	Gene of Interest
HVD	Hypervariable Domain
IIP	Innate Inhibiting Proteins
IVT RNA	In vitro transcribed RNA
LNP	Lipid Nanoparticle
nsPs	Non-structural proteins
PAMP	Pathogen-Associated Molecular Pattern
PRR	Pattern recognition receptor
RdRp	RNA-dependent RNA polymerase
RNAPII	RNA polymerase II
RRV	Ross River virus
saRNA	Self-amplifying RNA
SFV	Semliki Forest virus
SIE	Superinfection exclusion
SINV	Sindbis virus
ssRNA	Single-stranded RNA
UTR	Untranslated region
VEEV	Venezuelan Equine Encephalitis virus
WEEV	Western Equine Encephalitis virus

## References

[1] Dolgin E. (2021). The tangled history of mRNA vaccines. Nature.

[2] Wolff J.A., Malone R.W., Williams P., Chong W., Acsadi G., Jani A., Felgner P.L. (1990). Direct Gene Transfer into Mouse Muscle In Vivo. Science.

[3] Karikó K., Muramatsu H., Welsh F.A., Ludwig J., Kato H., Akira S., Weissman D. (2008). Incorporation of Pseudouridine Into mRNA Yields Superior Nonimmunogenic Vector with Increased Translational Capacity and Biological Stability. Mol. Ther..

[4] Hou X., Zaks T., Langer R., Dong Y. (2021). Lipid nanoparticles for mRNA delivery. Nat. Rev. Mater..

[5] Polack F.P., Thomas S.J., Kitchin N., Absalon J., Gurtman A., Lockhart S., Perez J.L., Pérez Marc G., Moreira E.D., Zerbini C. (2020). Safety and Efficacy of the BNT162b2 mRNA Covid-19 Vaccine. N. Engl. J. Med..

[6] El Sahly H.M., Baden L.R., Essink B., Doblecki-Lewis S., Martin J.M., Anderson E.J., Campbell T.B., Clark J., Jackson L.A., Fichtenbaum C.J. (2021). Efficacy of the mRNA-1273 SARS-CoV-2 Vaccine at Completion of Blinded Phase. N. Engl. J. Med..

[7] Vasileva O., Zaborova O., Shmykov B., Ivanov R., Reshetnikov V. (2024). Composition of lipid nanoparticles for targeted delivery: Application to mRNA therapeutics. Front. Pharmacol..

[8] Khlebnikova A., Kirshina A., Zakharova N., Ivanov R., Reshetnikov V. (2024). Current Progress in the Development of mRNA Vaccines Against Bacterial Infections. Int. J. Mol. Sci..

[9] Żak M.M., Zangi L. (2025). Clinical development of therapeutic mRNA applications. Mol. Ther..

[10] Muslimov A., Tereshchenko V., Shevyrev D., Rogova A., Lepik K., Reshetnikov V., Ivanov R. (2023). The Dual Role of the Innate Immune System in the Effectiveness of mRNA Therapeutics. Int. J. Mol. Sci..

[11] Moghimi S.M., Simberg D. (2022). Pro-inflammatory concerns with lipid nanoparticles. Mol. Ther..

[12] Barmada A., Klein J., Ramaswamy A., Brodsky N.N., Jaycox J.R., Sheikha H., Jones K.M., Habet V., Campbell M., Sumida T.S. (2023). Cytokinopathy with aberrant cytotoxic lymphocytes and profibrotic myeloid response in SARS-CoV-2 mRNA vaccine—Associated myocarditis. Sci. Immunol..

[13] Chen J., Chen J., Xu Q. (2022). Current Developments and Challenges of mRNA Vaccines. Annu. Rev. Biomed. Eng..

[14] Zhou W., Jiang L., Liao S., Wu F., Yang G., Hou L., Liu L., Pan X., Jia W., Zhang Y. (2023). Vaccines’ New Era-RNA Vaccine. Viruses.

[15] Maruggi G., Mallett C.P., Westerbeck J.W., Chen T., Lofano G., Friedrich K., Qu L., Sun J.T., McAuliffe J., Kanitkar A. (2022). A self-amplifying mRNA SARS-CoV-2 vaccine candidate induces safe and robust protective immunity in preclinical models. Mol. Ther..

[16] Pateev I., Seregina K., Ivanov R., Reshetnikov V. (2023). Biodistribution of RNA Vaccines and of Their Products: Evidence from Human and Animal Studies. Biomedicines.

[17] Gong Y., Yong D., Liu G., Xu J., Ding J., Jia W. (2024). A Novel Self-Amplifying mRNA with Decreased Cytotoxicity and Enhanced Protein Expression by Macrodomain Mutations. Adv. Sci..

[18] Arcturus Therapeutics and CSL Announce European Medicines Agency Validates Marketing Authorization Application for ARCT-154 Vaccine to Prevent COVID-19. https://ir.arcturusrx.com/news-releases/news-release-details/arcturus-therapeutics-and-csl-announce-european-medicines-agency.

[19] Vanluchene H., Gillon O., Peynshaert K., De Smedt S.C., Sanders N., Raemdonck K., Remaut K. (2024). Less is more: Self-amplifying mRNA becomes self-killing upon dose escalation in immune-competent retinal cells. Eur. J. Pharm. Biopharm..

[20] Minnaert A.-K., Vanluchene H., Verbeke R., Lentacker I., De Smedt S.C., Raemdonck K., Sanders N.N., Remaut K. (2021). Strategies for controlling the innate immune activity of conventional and self-amplifying mRNA therapeutics: Getting the message across. Adv. Drug Deliv. Rev..

[21] Casmil I.C., Jin J., Won E.-J., Huang C., Liao S., Cha-Molstad H., Blakney A.K. (2025). The advent of clinical self-amplifying RNA vaccines. Mol. Ther..

[22] Stokes A., Pion J., Binazon O., Laffont B., Bigras M., Dubois G., Blouin K., Young J.K., Ringenberg M.A., Ben Abdeljelil N. (2020). Nonclinical safety assessment of repeated administration and biodistribution of a novel rabies self-amplifying mRNA vaccine in rats. Regul. Toxicol. Pharmacol..

[23] Donahue D.A., Ballesteros C., Maruggi G., Glover C., Ringenberg M.A., Marquis M., Ben Abdeljelil N., Ashraf A., Rodriguez L.-A., Stokes A.H. (2023). Nonclinical Safety Assessment of Lipid Nanoparticle-and Emulsion-Based Self-Amplifying mRNA Vaccines in Rats. Int. J. Toxicol..

[24] Wang Z., Chen Y., Wu H., Wang M., Mao L., Guo X., Zhu J., Ye Z., Luo X., Yang X. (2024). Intravenous administration of IL-12 encoding self-replicating RNA-lipid nanoparticle complex leads to safe and effective antitumor responses. Sci. Rep..

[25] McGill M.R. (2016). The past and present of serum aminotransferases and the future of liver injury biomarkers. EXCLI J..

[26] Lawson J.A., Fisher M.A., Simmons C.A., Farhood A., Jaeschke H. (1998). Parenchymal cell apoptosis as a signal for sinusoidal sequestration and transendothelial migration of neutrophils in murine models of endotoxin and fas-antibody-induced liver injury. Hepatology.

[27] Bajt M.L. (2000). Protection against Fas Receptor-Mediated Apoptosis in Hepatocytes and Nonparenchymal Cells by a Caspase-8 Inhibitor in Vivo: Evidence for a Postmitochondrial Processing of Caspase-8. Toxicol. Sci..

[28] Low J.G., De Alwis R., Chen S., Kalimuddin S., Leong Y.S., Mah T.K.L., Yuen N., Tan H.C., Zhang S.L., Sim J.X.Y. (2022). A phase I/II randomized, double-blinded, placebo-controlled trial of a self-amplifying Covid-19 mRNA vaccine. Npj Vaccines.

[29] Flanagan K.L., Fink A.L., Plebanski M., Klein S.L. (2017). Sex and Gender Differences in the Outcomes of Vaccination over the Life Course. Annu. Rev. Cell Dev. Biol..

[30] Wojcechowskyj J.A., Jong R.M., Mäger I., Flach B., Munson P.V., Mukherjee P.P., Mertins B., Barcay K.R., Folliard T. (2025). Controlling reactogenicity while preserving immunogenicity from a self-amplifying RNA vaccine by modulating nucleocytoplasmic transport. Npj Vaccines.

[31] Spikevax-Previously-COVID-19-Vaccine-Moderna-h-c-5791-ii-42-Epar-Assessment-Report-Variation_en. https://www.ema.europa.eu/en/medicines/human/EPAR/spikevax.

[32] Reshetnikov V., Shepelkova G., Rybakova A., Trashkov A., Yeremeev V., Ivanov R. (2024). The candidate anti-tuberculosis mRNA vaccine immunogenicity and reactogenicity dependency on the animal’s sex and the vaccine dose. Bull. Russ. State Med. Univ..

[33] Jackson L.A., Anderson E.J., Rouphael N.G., Roberts P.C., Makhene M., Coler R.N., McCullough M.P., Chappell J.D., Denison M.R., Stevens L.J. (2020). An mRNA Vaccine against SARS-CoV-2—Preliminary Report. N. Engl. J. Med..

[34] Walsh E.E., Frenck R.W., Falsey A.R., Kitchin N., Absalon J., Gurtman A., Lockhart S., Neuzil K., Mulligan M.J., Bailey R. (2020). Safety and Immunogenicity of Two RNA-Based Covid-19 Vaccine Candidates. N. Engl. J. Med..

[35] Jin Z., Wu J., Wang Y., Huang T., Zhao K., Liu J., Wang H., Zhu T., Gou J., Huang H. (2023). Safety and immunogenicity of the COVID-19 mRNA vaccine CS-2034: A randomized, double-blind, dose-exploration, placebo-controlled multicenter Phase I clinical trial in healthy Chinese adults. J. Infect..

[36] Pollock K.M., Cheeseman H.M., Szubert A.J., Libri V., Boffito M., Owen D., Bern H., McFarlane L.R., O’Hara J., Lemm N.-M. (2022). Safety and immunogenicity of a self-amplifying RNA vaccine against COVID-19: COVAC1, a phase I, dose-ranging trial. eClinicalMedicine.

[37] Szubert A.J., Pollock K.M., Cheeseman H.M., Alagaratnam J., Bern H., Bird O., Boffito M., Byrne R., Cole T., Cosgrove C.A. (2023). COVAC1 phase 2a expanded safety and immunogenicity study of a self-amplifying RNA vaccine against SARS-CoV-2. eClinicalMedicine.

[38] Hồ N.T., Hughes S.G., Ta V.T., Phan L.T., Đỗ Q., Nguyễn T.V., Phạm A.T.V., Thị Ngọc Đặng M., Nguyễn L.V., Trịnh Q.V. (2024). Safety, immunogenicity and efficacy of the self-amplifying mRNA ARCT-154 COVID-19 vaccine: Pooled phase 1, 2, 3a and 3b randomized, controlled trials. Nat. Commun..

[39] Maine C.J., Miyake-Stoner S.J., Spasova D.S., Picarda G., Chou A.C., Brand E.D., Olesiuk M.D., Domingo C.C., Little H.J., Goodman T.T. (2025). Safety and immunogenicity of an optimized self-replicating RNA platform for low dose or single dose vaccine applications: A randomized, open label Phase I study in healthy volunteers. Nat. Commun..

[40] Kitonsa J., Serwanga J., Cheeseman H.M., Abaasa A., Lunkuse J.F., Ruzagira E., Kato L., Nambaziira F., Oluka G.K., Gombe B. (2025). Safety and Immunogenicity of a Modified Self-Amplifying Ribonucleic Acid (saRNA) Vaccine Encoding SARS-CoV-2 Spike Glycoprotein in SARS-CoV-2 Seronegative and Seropositive Ugandan Individuals. Vaccines.

[41] Saraf A., Gurjar R., Kaviraj S., Kulkarni A., Kumar D., Kulkarni R., Virkar R., Krishnan J., Yadav A., Baranwal E. (2024). An Omicron-specific, self-amplifying mRNA booster vaccine for COVID-19: A phase 2/3 randomized trial. Nat. Med..

[42] Ndeupen S., Qin Z., Jacobsen S., Bouteau A., Estanbouli H., Igyártó B.Z. (2021). The mRNA-LNP platform’s lipid nanoparticle component used in preclinical vaccine studies is highly inflammatory. iScience.

[43] Korzun T., Moses A.S., Jozic A., Grigoriev V., Newton S., Kim J., Diba P., Sattler A., Levasseur P.R., Le N. (2024). Lipid Nanoparticles Elicit Reactogenicity and Sickness Behavior in Mice Via Toll-Like Receptor 4 and Myeloid Differentiation Protein 88 Axis. ACS Nano.

[44] Kang D.D., Hou X., Wang L., Xue Y., Li H., Zhong Y., Wang S., Deng B., McComb D.W., Dong Y. (2024). Engineering LNPs with polysarcosine lipids for mRNA delivery. Bioact. Mater..

[45] Strauss J.H., Strauss E.G. (1994). The alphaviruses: Gene expression, replication, and evolution. Microbiol. Rev..

[46] Kulasegaran-Shylini R., Atasheva S., Gorenstein D.G., Frolov I. (2009). Structural and Functional Elements of the Promoter Encoded by the 5′ Untranslated Region of the Venezuelan Equine Encephalitis Virus Genome. J. Virol..

[47] Frolov I., Hardy R., Rice C.M. (2001). Cis-acting RNA elements at the 5′ end of Sindbis virus genome RNA regulate minus- and plus-strand RNA synthesis. RNA.

[48] Michel G., Petrakova O., Atasheva S., Frolov I. (2007). Adaptation of Venezuelan equine encephalitis virus lacking 51-nt conserved sequence element to replication in mammalian and mosquito cells. Virology.

[49] Shirako Y., Strauss J.H. (1994). Regulation of Sindbis virus RNA replication: Uncleaved P123 and nsP4 function in minus-strand RNA synthesis, whereas cleaved products from P123 are required for efficient plus-strand RNA synthesis. J. Virol..

[50] Lemm J.A., Rümenapf T., Strauss E.G., Strauss J.H., Rice C.M. (1994). Polypeptide requirements for assembly of functional Sindbis virus replication complexes: A model for the temporal regulation of minus- and plus-strand RNA synthesis. EMBO J..

[51] Rupp J.C., Sokoloski K.J., Gebhart N.N., Hardy R.W. (2015). Alphavirus RNA synthesis and non-structural protein functions. J. Gen. Virol..

[52] Tan Y.B., Chmielewski D., Law M.C.Y., Zhang K., He Y., Chen M., Jin J., Luo D. (2022). Molecular architecture of the Chikungunya virus replication complex. Sci. Adv..

[53] Laurent T., Kumar P., Liese S., Zare F., Jonasson M., Carlson A., Carlson L.-A. (2022). Architecture of the chikungunya virus replication organelle. eLife.

[54] Ahola T., Kääriäinen L. (1995). Reaction in alphavirus mRNA capping: Formation of a covalent complex of nonstructural protein nsP1 with 7-methyl-GMP. Proc. Natl. Acad. Sci. USA.

[55] Vasiljeva L., Merits A., Auvinen P., Kääriäinen L. (2000). Identification of a Novel Function of the AlphavirusCapping Apparatus. J. Biol. Chem..

[56] Rausalu K., Utt A., Quirin T., Varghese F.S., Žusinaite E., Das P.K., Ahola T., Merits A. (2016). Chikungunya virus infectivity, RNA replication and non-structural polyprotein processing depend on the nsP2 protease’s active site cysteine residue. Sci. Rep..

[57] Rikkonen M., Peränen J., Kääriäinen L. (1994). ATPase and GTPase activities associated with Semliki Forest virus nonstructural protein nsP2. J. Virol..

[58] Karpe Y.A., Aher P.P., Lole K.S. (2011). NTPase and 5′-RNA Triphosphatase Activities of Chikungunya Virus nsP2 Protein. PLoS ONE.

[59] Das P.K., Merits A., Lulla A. (2014). Functional Cross-talk between Distant Domains of Chikungunya Virus Non-structural Protein 2 Is Decisive for Its RNA-modulating Activity. J. Biol. Chem..

[60] Akhrymuk I., Kulemzin S.V., Frolova E.I. (2012). Evasion of the Innate Immune Response: The Old World Alphavirus nsP2 Protein Induces Rapid Degradation of Rpb1, a Catalytic Subunit of RNA Polymerase II. J. Virol..

[61] Meshram C.D., Phillips A.T., Lukash T., Shiliaev N., Frolova E.I., Frolov I. (2020). Mutations in Hypervariable Domain of Venezuelan Equine Encephalitis Virus nsP3 Protein Differentially Affect Viral Replication. J. Virol..

[62] Hick T.A.H., Zotler T., Bosveld D., Geertsema C., Van Oers M.M., Pijlman G.P. (2024). Venezuelan equine encephalitis virus non-structural protein 3 dictates superinfection exclusion in mammalian cells. Npj Viruses.

[63] Rubach J.K., Wasik B.R., Rupp J.C., Kuhn R.J., Hardy R.W., Smith J.L. (2009). Characterization of purified Sindbis virus nsP4 RNA-dependent RNA polymerase activity in vitro. Virology.

[64] Rupp J.C., Jundt N., Hardy R.W. (2011). Requirement for the Amino-Terminal Domain of Sindbis Virus nsP4 during Virus Infection. J. Virol..

[65] Tomar S., Hardy R.W., Smith J.L., Kuhn R.J. (2006). Catalytic Core of Alphavirus Nonstructural Protein nsP4 Possesses Terminal Adenylyltransferase Activity. J. Virol..

[66] Akhrymuk I., Lukash T., Frolov I., Frolova E.I. (2019). Novel Mutations in nsP2 Abolish Chikungunya Virus-Induced Transcriptional Shutoff and Make the Virus Less Cytopathic without Affecting Its Replication Rates. J. Virol..

[67] Frolov I., Garmashova N., Atasheva S., Frolova E.I. (2009). Random Insertion Mutagenesis of Sindbis Virus Nonstructural Protein 2 and Selection of Variants Incapable of Downregulating Cellular Transcription. J. Virol..

[68] Garmashova N., Atasheva S., Kang W., Weaver S.C., Frolova E., Frolov I. (2007). Analysis of Venezuelan Equine Encephalitis Virus Capsid Protein Function in the Inhibition of Cellular Transcription. J. Virol..

[69] Garmashova N., Gorchakov R., Volkova E., Paessler S., Frolova E., Frolov I. (2007). The Old World and New World Alphaviruses Use Different Virus-Specific Proteins for Induction of Transcriptional Shutoff. J. Virol..

[70] Petrakova O., Volkova E., Gorchakov R., Paessler S., Kinney R.M., Frolov I. (2005). Noncytopathic Replication of Venezuelan Equine Encephalitis Virus and Eastern Equine Encephalitis Virus Replicons in Mammalian Cells. J. Virol..

[71] Li Y., Teague B., Zhang Y., Su Z., Porter E., Dobosh B., Wagner T., Irvine D.J., Weiss R. (2019). In Vitro evolution of enhanced RNA replicons for immunotherapy. Sci. Rep..

[72] Maruggi G., Shaw C.A., Otten G.R., Mason P.W., Beard C.W. (2013). Engineered alphavirus replicon vaccines based on known attenuated viral mutants show limited effects on immunogenicity. Virology.

[73] Lin G., Zhang Y. (2023). Mutations in the non-structural protein coding region regulate gene expression from replicon RNAs derived from Venezuelan equine encephalitis virus. Biotechnol. Lett..

[74] Beitzel B.F., Bakken R.R., Smith J.M., Schmaljohn C.S. (2010). High-Resolution Functional Mapping of the Venezuelan Equine Encephalitis Virus Genome by Insertional Mutagenesis and Massively Parallel Sequencing. PLoS Pathog..

[75] Quintana V., Caillava J., Byk L.A., Mondotte J.A., Battini L., Tarte P., Samsa M.M., Filomatori C.V., Alvarez D.E. (2025). Improvement of the potency of a N1-methylpseudouridine-modified self-amplifying RNA through mutations in the RNA-dependent-RNA-polymerase. J. Biol. Chem..

[76] Chamberlain J., Dowall S.D., Smith J., Pearson G., Graham V., Raynes J., Hewson R. (2025). Attenuation of Chikungunya Virus by a Single Amino Acid Substitution in the nsP1 Component of a Non-Structural Polyprotein. Viruses.

[77] Utt A., Das P.K., Varjak M., Lulla V., Lulla A., Merits A., Perlman S. (2015). Mutations Conferring a Noncytotoxic Phenotype on Chikungunya Virus Replicons Compromise Enzymatic Properties of Nonstructural Protein 2. J. Virol..

[78] Garmashova N., Gorchakov R., Frolova E., Frolov I. (2006). Sindbis Virus Nonstructural Protein nsP2 Is Cytotoxic and Inhibits Cellular Transcription. J. Virol..

[79] Dominguez F., Palchevska O., Frolova E.I., Frolov I. (2023). Alphavirus-based replicons demonstrate different interactions with host cells and can be optimized to increase protein expression. J. Virol..

[80] Akhrymuk I., Frolov I., Frolova E.I. (2018). Sindbis Virus Infection Causes Cell Death by nsP2-Induced Transcriptional Shutoff or by nsP3-Dependent Translational Shutoff. J. Virol..

[81] Kafai N.M., Diamond M.S., Fox J.M. (2022). Distinct Cellular Tropism and Immune Responses to Alphavirus Infection. Annu. Rev. Immunol..

[82] Guerrero-Arguero I., Tellez-Freitas C.M., Weber K.S., Berges B.K., Robison R.A., Pickett B.E. (2021). Alphaviruses: Host pathogenesis, immune response, and vaccine & treatment updates. J. Gen. Virol..

[83] Leung J.Y.-S., Ng M.M.-L., Chu J.J.H. (2011). Replication of Alphaviruses: A Review on the Entry Process of Alphaviruses into Cells. Adv. Virol..

[84] Sun Z., Liu Y., Zhang H., Ge T., Pan Y., Liu Y., Wu M., Shan T., Zhu G., Wu Q. (2025). Next-Generation saRNA Platforms: Systematic Screening and Engineering Enhances Superior Protein Expression and Organ-Specific Targeting for RNA Therapeutics. bioRxiv.

[85] Morais P., Adachi H., Yu Y.-T. (2021). The Critical Contribution of Pseudouridine to mRNA COVID-19 Vaccines. Front. Cell Dev. Biol..

[86] Martinez N.M., Su A., Burns M.C., Nussbacher J.K., Schaening C., Sathe S., Yeo G.W., Gilbert W.V. (2022). Pseudouridine synthases modify human pre-mRNA co-transcriptionally and affect pre-mRNA processing. Mol. Cell.

[87] Svitkin Y.V., Cheng Y.M., Chakraborty T., Presnyak V., John M., Sonenberg N. (2017). N1-methyl-pseudouridine in mRNA enhances translation through eIF2α-dependent and independent mechanisms by increasing ribosome density. Nucleic Acids Res..

[88] Monroe J., Eyler D.E., Mitchell L., Deb I., Bojanowski A., Srinivas P., Dunham C.M., Roy B., Frank A.T., Koutmou K.S. (2024). N1-Methylpseudouridine and pseudouridine modifications modulate mRNA decoding during translation. Nat. Commun..

[89] Azizi H., Renner T.M., Agbayani G., Simard B., Dudani R., Harrison B.A., Iqbal U., Jia Y., McCluskie M.J., Akache B. (2024). Self-amplifying RNAs generated with the modified nucleotides 5-methylcytidine and 5-methyluridine mediate strong expression and immunogenicity in vivo. NAR Mol. Med..

[90] Miyazato P., Noguchi T., Ogawa F., Sugimoto T., Fauzyah Y., Sasaki R., Ebina H. (2024). 1mΨ influences the performance of various positive-stranded RNA virus-based replicons. Sci. Rep..

[91] Komori M., Morey A.L., Quiñones-Molina A.A., Fofana J., Romero L., Peters E., Matsuda K., Gummuluru S., Smith J.F., Akahata W. (2023). Incorporation of 5 methylcytidine alleviates innate immune response to self-amplifying RNA vaccine. bioRxiv.

[92] McGee J.E., Kirsch J.R., Kenney D., Cerbo F., Chavez E.C., Shih T.-Y., Douam F., Wong W.W., Grinstaff M.W. (2025). Complete substitution with modified nucleotides in self-amplifying RNA suppresses the interferon response and increases potency. Nat. Biotechnol..

[93] Aboshi M., Matsuda K., Kawakami D., Kono K., Kazami Y., Sekida T., Komori M., Morey A.L., Suga S., Smith J.F. (2024). Safety and immunogenicity of VLPCOV-02, a SARS-CoV-2 self-amplifying RNA vaccine with a modified base, 5-methylcytosine. iScience.

[94] Li D., Wu M. (2021). Pattern recognition receptors in health and diseases. Signal Transduct. Target. Ther..

[95] Kawai T., Akira S. (2006). TLR signaling. Cell Death Differ..

[96] Takeda K. (2004). Toll-like receptors in innate immunity. Int. Immunol..

[97] Kumar H., Kawai T., Akira S. (2009). Toll-like receptors and innate immunity. Biochem. Biophys. Res. Commun..

[98] Vercammen E., Staal J., Beyaert R. (2008). Sensing of Viral Infection and Activation of Innate Immunity by Toll-Like Receptor 3. Clin. Microbiol. Rev..

[99] Santoro M.G. (2003). NEW EMBO MEMBER’S REVIEW: NF-kappaB and virus infection: Who controls whom. EMBO J..

[100] Devoldere J., Dewitte H., De Smedt S.C., Remaut K. (2016). Evading innate immunity in nonviral mRNA delivery: Don’t shoot the messenger. Drug Discov. Today.

[101] Takeuchi O., Akira S. (2007). Recognition of viruses by innate immunity. Immunol. Rev..

[102] Yoneyama M., Fujita T. (2008). Structural Mechanism of RNA Recognition by the RIG-I-like Receptors. Immunity.

[103] Barral P.M., Sarkar D., Su Z., Barber G.N., DeSalle R., Racaniello V.R., Fisher P.B. (2009). Functions of the cytoplasmic RNA sensors RIG-I and MDA-5: Key regulators of innate immunity. Pharmacol. Ther..

[104] Tregoning J.S., Stirling D.C., Wang Z., Flight K.E., Brown J.C., Blakney A.K., McKay P.F., Cunliffe R.F., Murugaiah V., Fox C.B. (2023). Formulation, inflammation, and RNA sensing impact the immunogenicity of self-amplifying RNA vaccines. Mol. Ther. Nucleic Acids.

[105] Ueta M., Hamuro J., Kiyono H., Kinoshita S. (2005). Triggering of TLR3 by polyI:C in human corneal epithelial cells to induce inflammatory cytokines. Biochem. Biophys. Res. Commun..

[106] Nikonov A., Mölder T., Sikut R., Kiiver K., Männik A., Toots U., Lulla A., Lulla V., Utt A., Merits A. (2013). RIG-I and MDA-5 Detection of Viral RNA-dependent RNA Polymerase Activity Restricts Positive-Strand RNA Virus Replication. PLoS Pathog..

[107] Akhrymuk I., Frolov I., Frolova E.I. (2016). Both RIG-I and MDA5 detect alphavirus replication in concentration-dependent mode. Virology.

[108] McDougal M.B., De Maria A.M., Ohlson M.B., Kumar A., Xing C., Schoggins J.W. (2023). Interferon inhibits a model RNA virus via a limited set of inducible effector genes. EMBO Rep..

[109] Rutherford M.N., Hannigan G.E., Williams B.R. (1988). Interferon-induced binding of nuclear factors to promoter elements of the 2-5A synthetase gene. EMBO J..

[110] Dey M., Cao C., Dar A.C., Tamura T., Ozato K., Sicheri F., Dever T.E. (2005). Mechanistic Link between PKR Dimerization, Autophosphorylation, and eIF2α Substrate Recognition. Cell.

[111] García M.A., Meurs E.F., Esteban M. (2007). The dsRNA protein kinase PKR: Virus and cell control. Biochimie.

[112] Corbet G.A., Burke J.M., Bublitz G.R., Tay J.W., Parker R. (2022). dsRNA-induced condensation of antiviral proteins modulates PKR activity. Proc. Natl. Acad. Sci. USA.

[113] Yu H., Megawati D., Zheng C., Rothenburg S., Zheng C. (2025). Protein Kinase R (PKR) as a Novel dsRNA Sensor in Antiviral Innate Immunity. Antiviral Innate Immunity.

[114] Balachandran S., Kim C.N., Yeh W.-C., Mak T.W., Bhalla K., Barber G.N. (1998). Activation of the dsRNA-dependent protein kinase, PKR, induces apoptosis through FADD-mediated death signaling. EMBO J..

[115] Thapa R.J., Nogusa S., Chen P., Maki J.L., Lerro A., Andrake M., Rall G.F., Degterev A., Balachandran S. (2013). Interferon-induced RIP1/RIP3-mediated necrosis requires PKR and is licensed by FADD and caspases. Proc. Natl. Acad. Sci. USA.

[116] Dahal B., Lin S.C., Carey B.D., Jacobs J.L., Dinman J.D., van Hoek M.L., Adams A.A., Kehn-Hall K. (2020). EGR1 upregulation following Venezuelan equine encephalitis virus infection is regulated by ERK and PERK pathways contributing to cell death. Virology.

[117] Harding H.P., Zhang Y., Ron D. (1999). Protein translation and folding are coupled by an endoplasmic-reticulum-resident kinase. Nature.

[118] Holm L. (1995). DNA polymerase β belongs to an ancient nucleotidyltransferase superfamily. Trends Biochem. Sci..

[119] Kristiansen H., Gad H.H., Eskildsen-Larsen S., Despres P., Hartmann R. (2011). The Oligoadenylate Synthetase Family: An Ancient Protein Family with Multiple Antiviral Activities. J. Interferon Cytokine Res..

[120] Ibsen M.S., Gad H.H., Andersen L.L., Hornung V., Julkunen I., Sarkar S.N., Hartmann R. (2015). Structural and functional analysis reveals that human OASL binds dsRNA to enhance RIG-I signaling. Nucleic Acids Res..

[121] Tanaka N., Nakanishi M., Kusakabe Y., Goto Y., Kitade Y., Nakamura K.T. (2004). Structural basis for recognition of 2′,5′-linked oligoadenylates by human ribonuclease L. EMBO J..

[122] Zhou A. (1993). Expression cloning of 2-5A-dependent RNAase: A uniquely regulated mediator of interferon action. Cell.

[123] Bisbal C., Silverman R.H. (2007). Diverse functions of RNase L and implications in pathology. Biochimie.

[124] Kubota K., Nakahara K., Ohtsuka T., Yoshida S., Kawaguchi J., Fujita Y., Ozeki Y., Hara A., Yoshimura C., Furukawa H. (2004). Identification of 2′-Phosphodiesterase, Which Plays a Role in the 2-5A System Regulated by Interferon. J. Biol. Chem..

[125] Schmidt A., Chernajovsky Y., Shulman L., Federman P., Berissi H., Revel M. (1979). An interferon-induced phosphodiesterase degrading (2′–5′) oligoisoadenylate and the C-C-A terminus of tRNA. Proc. Natl. Acad. Sci. USA.

[126] Silverman R.H., Gilbert C.S., Kerr I.M., Wreschner D.H. (2005). Synthesis, Characterization and Properties of ppp(A2′p)nApCp and Related High-Specific-Activity 32P-Labelled Derivatives of ppp(A2′p)nA. Eur. J. Biochem..

[127] Takata M.A., Gonçalves-Carneiro D., Zang T.M., Soll S.J., York A., Blanco-Melo D., Bieniasz P.D. (2017). CG dinucleotide suppression enables antiviral defence targeting non-self RNA. Nature.

[128] Johnson B., VanBlargan L.A., Xu W., White J.P., Shan C., Shi P.-Y., Zhang R., Adhikari J., Gross M.L., Leung D.W. (2018). Human IFIT3 Modulates IFIT1 RNA Binding Specificity and Protein Stability. Immunity.

[129] Poleganov M.A., Eminli S., Beissert T., Herz S., Moon J.-I., Goldmann J., Beyer A., Heck R., Burkhart I., Barea Roldan D. (2015). Efficient Reprogramming of Human Fibroblasts and Blood-Derived Endothelial Progenitor Cells Using Nonmodified RNA for Reprogramming and Immune Evasion. Hum. Gene Ther..

[130] Ohto T., Konishi M., Tanaka H., Onomoto K., Yoneyama M., Nakai Y., Tange K., Yoshioka H., Akita H. (2019). Inhibition of the Inflammatory Pathway Enhances Both the *In Vitro* and *In Vivo* Transfection Activity of Exogenous *in Vitro*-Transcribed mRNAs Delivered by Lipid Nanoparticles. Biol. Pharm. Bull..

[131] Devasthanam A.S. (2014). Mechanisms underlying the inhibition of interferon signaling by viruses. Virulence.

[132] Liu Y., Chin J.M., Choo E.L., Phua K.K.L. (2019). Messenger RNA translation enhancement by immune evasion proteins: A comparative study between EKB (vaccinia virus) and NS1 (influenza A virus). Sci. Rep..

[133] Beissert T., Koste L., Perkovic M., Walzer K.C., Erbar S., Selmi A., Diken M., Kreiter S., Türeci Ö., Sahin U. (2017). Improvement of *In Vivo* Expression of Genes Delivered by Self-Amplifying RNA Using Vaccinia Virus Immune Evasion Proteins. Hum. Gene Ther..

[134] Perdiguero B., Esteban M. (2009). The Interferon System and Vaccinia Virus Evasion Mechanisms. J. Interferon Cytokine Res..

[135] Krug R.M. (2015). Functions of the influenza A virus NS1 protein in antiviral defense. Curr. Opin. Virol..

[136] Hale B.G., Randall R.E., Ortín J., Jackson D. (2008). The multifunctional NS1 protein of influenza A viruses. J. Gen. Virol..

[137] Blakney A.K., McKay P.F., Bouton C.R., Hu K., Samnuan K., Shattock R.J. (2021). Innate Inhibiting Proteins Enhance Expression and Immunogenicity of Self-Amplifying RNA. Mol. Ther..

[138] Blakney A.K., Zhu Y., McKay P.F., Bouton C.R., Yeow J., Tang J., Hu K., Samnuan K., Grigsby C.L., Shattock R.J. (2020). Big Is Beautiful: Enhanced saRNA Delivery and Immunogenicity by a Higher Molecular Weight, Bioreducible, Cationic Polymer. ACS Nano.

[139] Borghese F., Sorgeloos F., Cesaro T., Michiels T. (2019). The Leader Protein of Theiler’s Virus Prevents the Activation of PKR. J. Virol..

[140] Pepini T., Pulichino A.-M., Carsillo T., Carlson A.L., Sari-Sarraf F., Ramsauer K., Debasitis J.C., Maruggi G., Otten G.R., Geall A.J. (2017). Induction of an IFN-Mediated Antiviral Response by a Self-Amplifying RNA Vaccine: Implications for Vaccine Design. J. Immunol..

[141] De Beuckelaer A., Pollard C., Van Lint S., Roose K., Van Hoecke L., Naessens T., Udhayakumar V.K., Smet M., Sanders N., Lienenklaus S. (2016). Type I Interferons Interfere with the Capacity of mRNA Lipoplex Vaccines to Elicit Cytolytic T Cell Responses. Mol. Ther..

[142] Zhong Z., Portela Catani J.P., Mc Cafferty S., Couck L., van Den Broeck W., Gorlé N., Vandenbroucke R.E., Devriendt B., Ulbert S., Cnops L. (2019). Immunogenicity and Protection Efficacy of a Naked Self-Replicating mRNA-Based Zika Virus Vaccine. Vaccines.

[143] Van Hoecke L., Roose K., Ballegeer M., Zhong Z., Sanders N.N., De Koker S., Saelens X., Van Lint S. (2020). The Opposing Effect of Type I IFN on the T Cell Response by Non-modified mRNA-Lipoplex Vaccines Is Determined by the Route of Administration. Mol. Ther. Nucleic Acids.

[144] McKay P.F., Hu K., Blakney A.K., Samnuan K., Brown J.C., Penn R., Zhou J., Bouton C.R., Rogers P., Polra K. (2020). Self-amplifying RNA SARS-CoV-2 lipid nanoparticle vaccine candidate induces high neutralizing antibody titers in mice. Nat. Commun..

[145] Jureka A.S., Kleinpeter A.B., Tipper J.L., Harrod K.S., Petit C.M. (2020). The influenza NS1 protein modulates RIG-I activation via a strain-specific direct interaction with the second CARD of RIG-I. J. Biol. Chem..

[146] Xing J., Wang S., Lin R., Mossman K.L., Zheng C. (2012). Herpes Simplex Virus 1 Tegument Protein US11 Downmodulates the RLR Signaling Pathway via Direct Interaction with RIG-I and MDA-5. J. Virol..

[147] Childs K., Randall R., Goodbourn S. (2012). Paramyxovirus V Proteins Interact with the RNA Helicase LGP2 To Inhibit RIG-I-Dependent Interferon Induction. J. Virol..

[148] Yang Y., Ye F., Zhu N., Wang W., Deng Y., Zhao Z., Tan W. (2015). Middle East respiratory syndrome coronavirus ORF4b protein inhibits type I interferon production through both cytoplasmic and nuclear targets. Sci. Rep..

